# Deciphering Resistome in Patients With Chronic Obstructive Pulmonary Diseases and *Clostridioides difficile* Infections

**DOI:** 10.3389/fmicb.2022.919907

**Published:** 2022-08-02

**Authors:** Youna Cho, Jieun Kim, Hyunjoo Pai, Mina Rho

**Affiliations:** ^1^Department of Computer Science, Hanyang University, Seoul, South Korea; ^2^Department of Internal Medicine, College of Medicine, Hanyang University, Seoul, South Korea; ^3^Department of Biomedical Informatics, Hanyang University, Seoul, South Korea

**Keywords:** mobilome, gut microbiome, antibiotics, *Clostridioides difficile* infections, chronic obstructive pulmonary diseases, antibiotic resistance genes

## Abstract

Antibiotics alter the gut microbiome and cause dysbiosis leading to antibiotic-resistant organisms. Different patterns of antibiotic administration cause a difference in bacterial composition and resistome in the human gut. We comprehensively investigated the association between the distribution of antibiotic resistance genes (ARGs), bacterial composition, and antibiotic treatments in patients with chronic obstructive pulmonary diseases (COPD) and *Clostridioides difficile* infections (CDI) who had chronic or acute intermittent use of antibiotics and compared them with healthy individuals. We analyzed the gut microbiomes of 61 healthy individuals, 16 patients with COPD, and 26 patients with CDI. The COPD patients were antibiotic-free before stool collection for a median of 40 days (Q1: 9.5; Q3: 60 days), while the CDI patients were antibiotic-free for 0 days (Q1: 0; Q3: 0.3). The intra-group beta diversity measured by the median Bray-Curtis index was the lowest for the healthy individuals (0.55), followed by the COPD (0.69) and CDI groups (0.72). The inter-group beta diversity was the highest among the healthy and CDI groups (median index = 0.89). The abundance of ARGs measured by the number of reads per kilobase per million reads (RPKM) was 684.2; 1,215.2; and 2,025.1 for the healthy, COPD, and CDI groups. It was negatively correlated with the alpha diversity of bacterial composition. For the prevalent ARG classes, healthy individuals had the lowest diversity and abundance of aminoglycoside, β-lactam, and macrolide-lincosamide-streptogramin (MLS) resistance genes, followed by the COPD and CDI groups. The abundances of *Enterococcus* and *Escherichia* species were positively correlated with ARG abundance and the days of antibiotic treatment, while *Bifidobacterium* and *Ruminococcus* showed negative correlations for the same. In addition, we analyzed the mobilome patterns of aminoglycoside and β-lactam resistance gene carriers using metagenomic sequencing data. In conclusion, the ARGs were significantly enhanced in the CDI and COPD groups than in healthy individuals. In particular, aminoglycoside and β-lactam resistance genes were more abundant in the CDI and COPD groups, but the dominant mobile genetic elements that enable the transfer of such genes showed similar prevalence patterns among the groups.

## Introduction

Antibiotic resistance is a serious threat to public health. With the indiscriminate use of antibiotics in humans and livestock, more antibiotic-resistant pathogenic bacteria have been reported (Canton and Coque, 2006; Mathew et al., 2007; MacGowan and Macnaughton, 2017; Peterson and Kaur, 2018; He, 2020). Multiple studies have shown that antibiotics change the bacterial composition in the gut (Chang et al., 2008; Seekatz et al., 2016; Palleja et al., 2018)—like the expansion of enterobacteria and depletion of butyrate-producing bacteria—and promote the development of multidrug-resistant strains (Magiorakos et al., 2012; Klemm et al., 2018).

Metagenomics-based computational methods can estimate the abundance of antibiotic resistance genes (ARGs) and profile their distribution in the gut microbiome (Hu et al., 2013; Forslund et al., 2014; Milani et al., 2016; Mac Aogáin et al., 2020). ARGs are identified using homology-based searches against ARG sequence databases, such as CARD (McArthur et al., 2013), ResFinder (Bortolaia et al., 2020), and ARG-ANNOT (Gupta et al., 2014), or machine learning-based models (Arango-Argoty et al., 2018). ARG-related mobilome and ARG origins have helped us understand their transfer mechanism (McMillan et al., 2019). Compared with the traditional polymerase chain reaction (PCR)-based methods to identify and profile ARGs, the metagenomic approach provides more comprehensive information about novel ARGs and the related mobilomes. It facilitates a deeper understanding of their dispersion, evolution, and effects on microbial communities.

Metagenomic sequencing data have been widely used to understand microbial communities and their association with the disease physiopathology (Hopkins and Macfarlane, 2002; Mammen and Sethi, 2016). Compared to non-CDI patients, the gut microbiome of CDI patients was enriched with *Enterococcus* but depleted with *Ruminococcus* and *Bifidobacterium* (Milani et al., 2016; Vincent et al., 2016; Amrane et al., 2019; Kim et al., 2020; Vasilescu et al., 2021). For COPD patients, there was a limited number of studies reporting the microbial composition in the gut. For example, a recent study reported that *Streptococcus,* and *Rotia* genera were enriched in the gut microbiome of stable COPD patients, while a 16S rRNA-based study found that the *Firmicutes* were enriched (Chiu et al., 2022). These metagenomic data have been used to find the association between ARGs and race, habitat, and antibiotic use by comparing their relative abundance and distribution patterns among different countries (Forslund et al., 2013; Hu et al., 2013; Yang et al., 2016). Several studies have revealed that antibiotics directly or indirectly affect the gut bacterial diversity (Dethlefsen and Relman, 2011; Pérez-Cobas et al., 2013; Buelow et al., 2017; Wang et al., 2020). Long-term antibiotic use may be associated with microbiota dysbiosis (Chang et al., 2008; Francino, 2015; Wang et al., 2020). A high frequency of horizontal transfer has also been reported in humans and their environments, suggesting the need for judicious use of antibiotics (Li et al., 2019; McMillan et al., 2019; He, 2020; Sun et al., 2020). Multi-habitat microbiomes have been analyzed in humans, animals, and external environments to determine how ARGs are shared among them (Pal et al., 2016).

This study compared the microbiomes and resistomes of three groups (two diseased and one healthy) using metagenomic sequencing data. We analyzed 103 Korean gut microbiome samples, including 61 healthy individuals, 16 patients with chronic obstructive pulmonary disease (COPD), and 26 patients with *C. difficile* infection (CDI). These groups have experienced different patterns of antibiotic treatment. The healthy individuals had minimum exposure to antibiotics, whereas the CDI patients were expected to have had recent heavy exposure to antibiotics. Patients with stable COPD were exposed to antibiotics intermittently due to COPD exacerbation. Our investigation revealed differences in microbial diversity among the three groups and the patterns of ARG prevalence and abundance. The association between the clinical characteristics and ARG abundance was analyzed. In addition, we identified the mobilome structures for the prevalent aminoglycoside resistance genes and β-lactamases (*bla*) in three groups and determined dominant patterns and their distribution. This study observed an association between antibiotic use, ARG distribution, and gut microbiome diversity using three groups that experienced different patterns of antibiotic treatment. In addition, dominant mobilome patterns of each ARG were observed in a population, regardless of the history of antibiotic use.

## Materials and Methods

### Enrolled Participants

The participants were divided into three groups: 61 healthy individuals, 16 COPD patients, and 26 CDI patients. The inclusion criteria for healthy adults were a zero score on Charlson’s comorbidity index and no admission history within the last year (Kim et al., 2020). The selected COPD patients had been followed up in an outpatient clinic under COPD diagnosis and prescribed antibiotics within the previous 2 years. The CDI patients who complained of unformed stool more than three times per day were selected, and the toxin gene was confirmed using multiplex PCR using *C. difficile* isolates from the patient (Kim et al., 2020). For healthy individuals and CDI patients, we used sequencing data obtained from a different study conducted by our group (Kim et al., 2020). For COPD patients, we sequenced the samples in this study. The collection and sequencing methods employed for COPD data in this study were the same as those used to obtain data for healthy individuals and CDI patients.

### Preparing Fecal DNA, Sequencing, and Filtering Sequences

We obtained 50 g of feces from 26 COPD patients and their medical records after informed consent. The feces were collected in a sterile container and stored at −80°C. About 2 ml of the specimen was used and total DNA was extracted using the Fast DNA SPIN Kit for Feces (MP Biomedicals, #116570200). Each sample was prepared according to the Illumina protocols. The quantification of DNA and the DNA quality were measured by PicoGreen and agarose gel electrophoresis. Briefly, 100 ng of genomic DNA for a 350 bp insert size is fragmented by Covaries. The fragmented DNA is blunt-ended and phosphorylated, following end repair. The appropriate library size is selected using different ratios of the sample purification beads. A single ‘A’ is ligated to the 3′ end, and Illumina adapters are then ligated to the fragments. The final ligated product is then quantified using qPCR according to the qPCR Quantification Protocol Guide and qualified using the Agilent Technologies 2,200 Tapestation (Agilent Technologies, Palo Alto CA, United States), HiSeq™ 4,000 platform (Illumina, San Diego, United States). The Illumina HiSeqX platform (Illumina, San Diego, United States) was used to sequence the samples. For each sample, all reads were 151-bp paired-end sequences with an insert size of ~350 bps. Quality trimming was performed using Sickle (Joshi, 2011) with Phred quality scores >20 and read length options >90 bp (pe -q 20 -t sanger -l 90). Reads containing ambiguous ‘N’ nucleotides were removed from the analysis.

### Determining Microbiota Composition

The microbiota composition of each sample was profiled using MetaPhlAn 2.0 (Segata et al., 2012). The genera with <0.1% abundance were filtered out from each sample, and those with >0.1% average abundance in any group were retained for analysis. To compare the microbiota of the healthy, COPD, and CDI groups, we analyzed alpha and beta diversity. Alpha diversity was measured using the Shannon index with genus abundance. Beta diversity was measured using the Bray–Curtis dissimilarity. The samples were clustered by microbiota composition using a Dirichlet multinomial mixture (DMM). Principal coordinates analysis (PCoA) was applied using the Bray–Curtis dissimilarity and visualized as a 2-dimensional scatter plot. PCoA results were color-labeled by groups (healthy individuals, CDI patients, and COPD patients) and DMM cluster results (*k* = 3).

### Identifying Antibiotic Resistance Genes

ARGs were identified using two different approaches: read mapping and gene sequence comparison. The read mapping approach measures the abundance of ARGs using the number of reads per kilobase per million mapped reads (RPKM). A total of 848 representative sequences were retained after clustering the ARGs obtained from the CARD 2.0.1 database (McArthur et al., 2013) using cd-hit (−c 0.9 −n 8; Li and Godzik, 2006) and manual curation (Kim et al., 2020). The reads were considered mapped if the similarity was >90% over 50% of the read length. An ARG was considered present if the reference coverage exceeded 70%. ARG abundance was expressed as $\log_{2}\left(RPKM+1\right)$. A gene sequence comparison approach was used to identify ARGs from contigs and reveal their mobilomes. The reads were assembled using MEGAHIT (Li et al., 2015) with –min-contig-len = 501. The genes were annotated from the assembled contigs using FragGeneScan (Rho et al., 2010) with -w 1 -t complete options. The annotated genes were considered ARG after comparing their protein sequences with the ones in CARD 2.0.1 (McArthur et al., 2013) using BLAST (Altschul et al., 1990). ARGs related to the efflux pumps and porins and those from uncultured bacteria were excluded from the analysis. The number of genes was counted and normalized by the million reads to give the gene counts per million reads (GPMR). We also used the gene counts per million genes (GPMG)—the ratio of ARGs to the genes in the microbiome.

### Identifying Mobilome Patterns in the Microbiomes

To determine the mobilome structure of ARGs, contig fragments harboring ARG and two neighboring genes upstream and downstream of the ARG were selected. These contigs were searched against a collection of ARGs and genes collected from the NCBI database for functional annotation. The matched position of the genome sequences was used as a reference to determine the mobilome structures in each sample. The following two criteria were used to assess the existence of each mobilome in the microbiome: (i) > 70% of the reference mobilome is covered by the sample reads, and (ii) there are one or more paired-end reads (i.e., conjunction reads) that are aligned both on the ARG and the neighboring gene. To be considered conjunction read, three criteria should be satisfied: (i) > 20% of each paired reads are aligned to either ARG or its neighboring gene (i.e., the rest are aligned to the intergenic regions), (ii) both paired reads have <10 bps of soft clips, and (iii) the distance between paired reads <350 × 3 bps.

### Statistical Analysis

SPSS version 26.0 for Windows (SPSS Inc., Armonk, NY, United States) was used to compare the demographics and clinical characteristics. A chi-square test was used to analyze categorical variables, and the Kruskal-Wallis test was used to analyze continuous variables. Spearman’s rank correlation test evaluated the relationship between the two variables. A *value of p* < 0.05, determined by a two-tailed test, was considered statistically significant. Shannon Index was calculated for analyzing alpha diversity and for the beta diversity function DistanceMatrix from skbio.stats.distance library was utilized using Bray–Curtis dissimilarity. To determine the correlation in a scatterplot, the geom_smooth function with the method = lm and formula = y ~ x option was used to build a linear line, and stat_cor with the “Pearson” method was used to get the r- and *value of p*.

## Results

### Demographic and Clinical Characteristics

We analyzed 61 healthy individuals, 16 COPD, and 26 CDI patients. Table 1 shows the demographic and clinical characteristics of each group. The females comprised 47.5% of the healthy group, 18.8% of the COPD group, and 50% of the CDI group. The median age of the healthy, COPD, and CDI groups was 46, 68, and 66.5 years, respectively. The Charlson Comorbidity Index score was significantly different (*p* < 0.001; Table 1; Supplementary Table S1). All patients with CDI were hospitalized, and all patients with stable COPD were enrolled in the outpatient clinic. Medical history within 60 days differed substantially between the groups. The median number of antibiotic-free days from the day of stool collection was 40 days (Q1: 9.5; Q3: 60) in COPD patients and 0 days (Q1: 0; Q3: 0.3) in CDI patients. The median days of antibiotic use within the last 60 days were 0, 8, and 18.5 days for the healthy, COPD, and CDI groups, respectively. Diverse broad-spectrum antibiotics were used more in the CDI patients than in COPD patients. In the COPD group, four patients (25%) received β-lactam, five (31%) fluoroquinolones, and one (6%) macrolide (Supplementary Table S2). In the CDI group, six patients (23%) received vancomycin, nine (35%) carbapenem, 20 (77%) β-lactam including β-lactam/β-lactamase inhibitor, seven (27%) fluoroquinolones, two (8%) doxycycline, six (23%) metronidazole, and one (4%) clindamycin, colistin, amikacin, or macrolide (Supplementary Table S3). The average DOT of antibiotics per group was higher in the CDI group (Table 1).

**Table 1:** Subject characteristics.

		Healthy (*N* = 61)	COPD (*N* = 16)	CDI (*N* = 26)	Value of *p*
Gender-female	*n* (%)	29 (47.5)	3 (18.8)	13 (50)	0.87
Age	median (1Q, 3Q)	46 (38.5, 50.5)	68 (61.5, 75)	66.5 (59.8, 76.3)	<0.001^*^
Body mass index	median (1Q, 3Q)	23.31 (21.58, 24.96)	20.9 (18.95, 25.6)	22.78 (19.25, 26.6)	0.503^*^
Charlson’s comorbidity score^+^	median (1Q, 3Q)	0 (0, 0)	3 (2.3, 3)	3 (1, 5)	<0.001^*^
Cerebrovascular disease	*n* (%)	0	2 (12.5)	8 (30.8)	<0.001
Dementia	*n* (%)	0	1 (6.3)	5 (19.2)	0.001
COPD	*n* (%)	0	16 (100)	5 (19.2)	0.001
Connective tissue disease	*n* (%)	0	1 (6.3)	4 (15.4)	0.002
Diabetes	*n* (%)	0	7 (43.8)	5 (19.2)	0.001
Moderate to severe renal disease	*n* (%)	0	2 (12.5)	9 (34.6)	<0.001
Past medical history within 60 days					
Admission - Yes	*n* (%)	0	3 (18.8)	16 (61.5)	<0.001
Antibiotics – Yes	*n* (%)	0 (0)	10 (62.5)	24 (92.3)	<0.001
Antibiotic-free days	median (1Q, 3Q)	60 (60, 60)	40 (9.5, 60)	0 (0, 0.3)	<0.001^*^
Days of antibiotic usage	median (1Q, 3Q)	0 (0, 0)	8 (0, 17.8)	18.5 (6.8, 41.5)	<0.001^*^
Probiotic - Yes	*n* (%)	12 (19.7)	7 (43.8)	5 (19.2)	0.754

Value of *p*, *p* for trend; COPD, chronic obstructive pulmonary disease; CDI, Clostridioides difficile infection; AIDS, acquired immunodeficiency syndrome.

* value of *p* by Kruskal-Wallis test.

### Microbial Distribution in CDI and COPD Patients and Healthy Individuals

**Figure 1:**
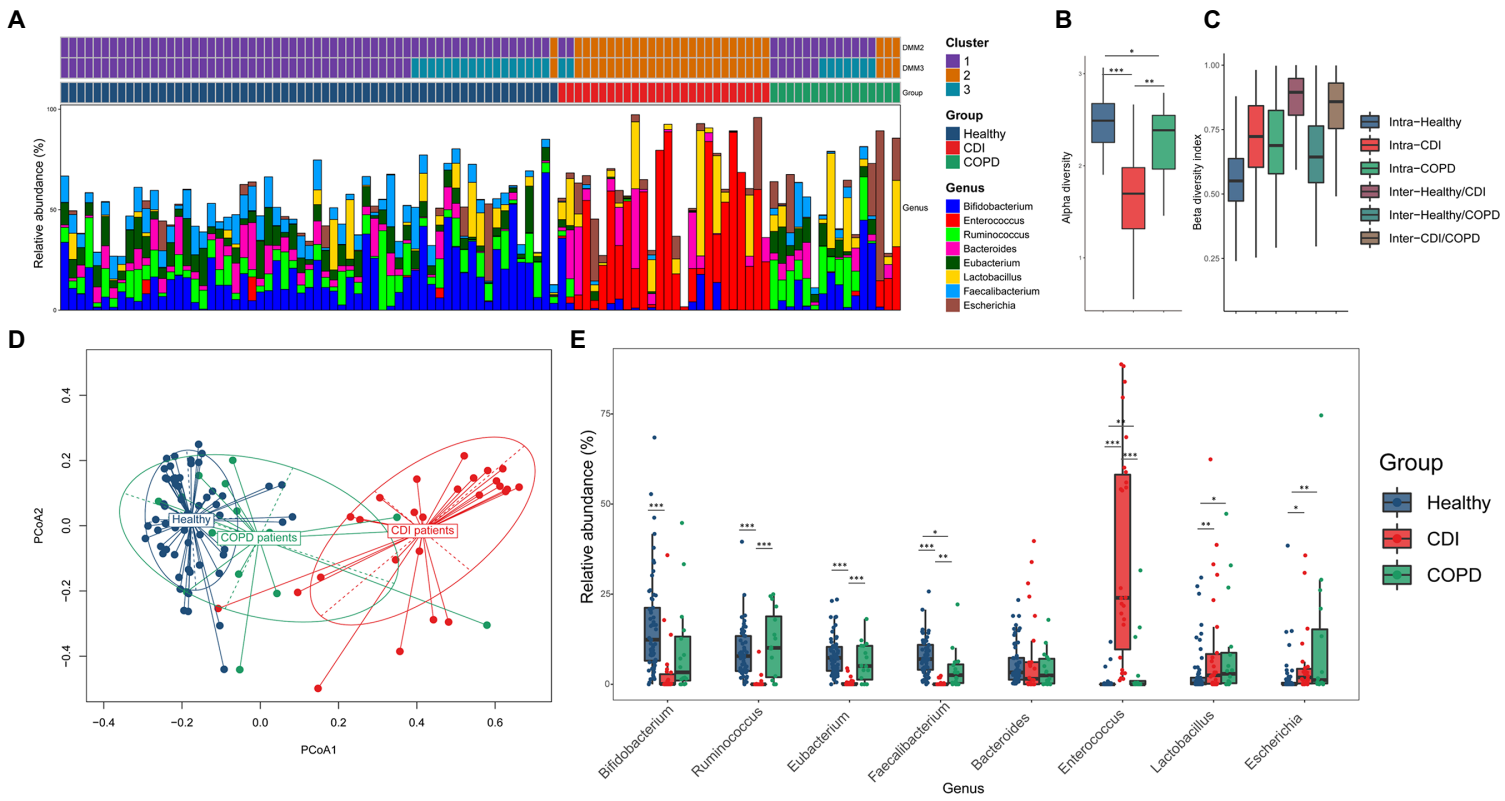
Bacterial composition in three groups. **(A)** The four most abundant taxa from each group (*Bifidobacterium*, *Ruminococcus*, *Eubacterium*, and *Faecalibacterium* in healthy; *Enterococcus*, *Lactobacillus*, *Escherichia*, and *Bacteroides* in CDI; *Ruminococcus*, *Eubacterium*, *Bifidobacterium*, and *Lactobacillus* in COPD) were visualized using stack bar, sorted by group and DMM clustering results (k = 2 and 3). The PCoA labeled by DMM clustering was provided in Supplementary Figure S2. **(B)** Alpha-diversity by Shannon index. **(C)** Beta-diversity by Bray–Curtis dissimilarity. For **(C)**, all pairs except intra-CDI and intra-COPD showed statistical significance (value of p <0.05). **(D)** PCoA of bacteria composition at the genus level, labeled by group, and **(E)** relative abundance of the prevalent genus in each group.

The microbial composition was compared among the three groups. The CDI and healthy groups showed distinct traits in the microbial composition, while the COPD samples showed both healthy and CDI traits (Figures 1A,D; Supplementary Figure S1). The most abundant genera were *Bifidobacterium*, *Ruminococcus*, and *Enterococcus* in the healthy, COPD, and CDI groups, respectively (Figures 1A,E). The median proportion of *Bifidobacterium* was 12.4% in the healthy group, 3.3% in COPD, and 0.1% in CDI (*p* < 0.01 for the healthy vs. CDI groups). Notably, the proportion of *Enterococcus* was significantly higher in the CDI group (24%) but low in the healthy (0.0%) and COPD groups (0.0%; *p* < 0.01). *Ruminococcus* was the most abundant genus in the COPD group (10.1%), had comparable levels in the healthy group (7.8%), but was scarce in the CDI group (0.0%; *p* < 0.01 between COPD vs. CDI and between healthy vs. CDI). The genera (mean proportion ≥ 0.1% in any group) and their mean proportion values in each group are listed in Supplementary Table S4. *Enterococcus* was significantly high in the CDI group, with a median proportion of 2.5% or below for all other genera. Such group-specific genus composition showed an association with antibiotic resistance composition, presented in the next section.

The distinctive pattern described above is represented in the principal coordinates analysis (PCoA) plot constructed with the relative abundance of each genus (Figure 1D). Samples of CDI and healthy individuals diverged in opposite directions. The samples of the COPD patients (81.3%) were closer to healthy samples; however, three COPD samples were found closer to the CDI group showing a high abundance of *Enterococcus* (13.1–31.6%; 0.0% for the median value in the COPD group). These three patients had received broad-spectrum antibiotics during the past 2 months. The one CDI sample is most similar to the healthy samples that harbored only 1.1% of *Enterococcus* (24.0% for the median value in the CDI group). The other three CDI samples closer to the healthy samples had less than or around 3% of *Enterococcus*.

To measure the diversity of genera in each group, alpha and beta diversity were used (Figures 1B,C). The CDI group had the lowest alpha diversity. The median Shannon indices for the healthy, COPD, and CDI groups were 2.5, 2.4, and 1.7, respectively. Notably, the microbial diversities in the CDI and COPD groups were lower than those in the healthy group. We measured the intra- and inter-group Bray-Curtis index to analyze the beta diversity between groups. The median Bray-Curtis index in healthy samples was the lowest, followed by the COPD and CDI groups (median index of 0.55, 0.69, and 0.72 for healthy, COPD, and CDI, respectively; Figure 1C). Like the observation in PCoA (Figure 1D), the inter-group beta diversity between healthy and COPD groups was the lowest (median index of 0.64), while that between the healthy and CDI groups was the highest (median index of 0.89), suggesting that the healthy and COPD groups had similar genus composition.

To further investigate the relationship between the bacterial composition and disease group, the Dirichlet multinomial mixture (DMM) clustering method was used to cluster the entire sample by genus proportion (Figure 1A; Supplementary Figures S2A,B). In the DMM model (*k* = 2 for the lowest Laplace approximation value), one cluster included most of the healthy and COPD samples (98% of healthy; 81% of COPD; see Cluster 1 in Figure 1A), while the other cluster included most of the CDI samples (92%), one healthy, and three COPD samples (Cluster 2 in Figure 1A). But when the samples were clustered into three clusters (*k* = 3 for the second-lowest Laplace approximation value), the third cluster separated some healthy and COPD samples from Cluster 1 (see Cluster 3 in Figure 1A; Supplementary Figure S2B): 28% of healthy and 44% of COPD samples. We found that Cluster 1 had similar proportions of *Bifidobacterium*, *Ruminococcus*, *Eubacterium*, and *Faecalibacterium* (median values between 8.1 and 9.2%), while Cluster 3 had a higher proportion of *Bifidobacterium* and lower *Bacteroides* than Cluster 1 (Supplementary Figure S2B and Table S4).

### Antibiotic Resistance Genes in CDI and COPD Patients and Healthy Individuals

**Figure 2:**
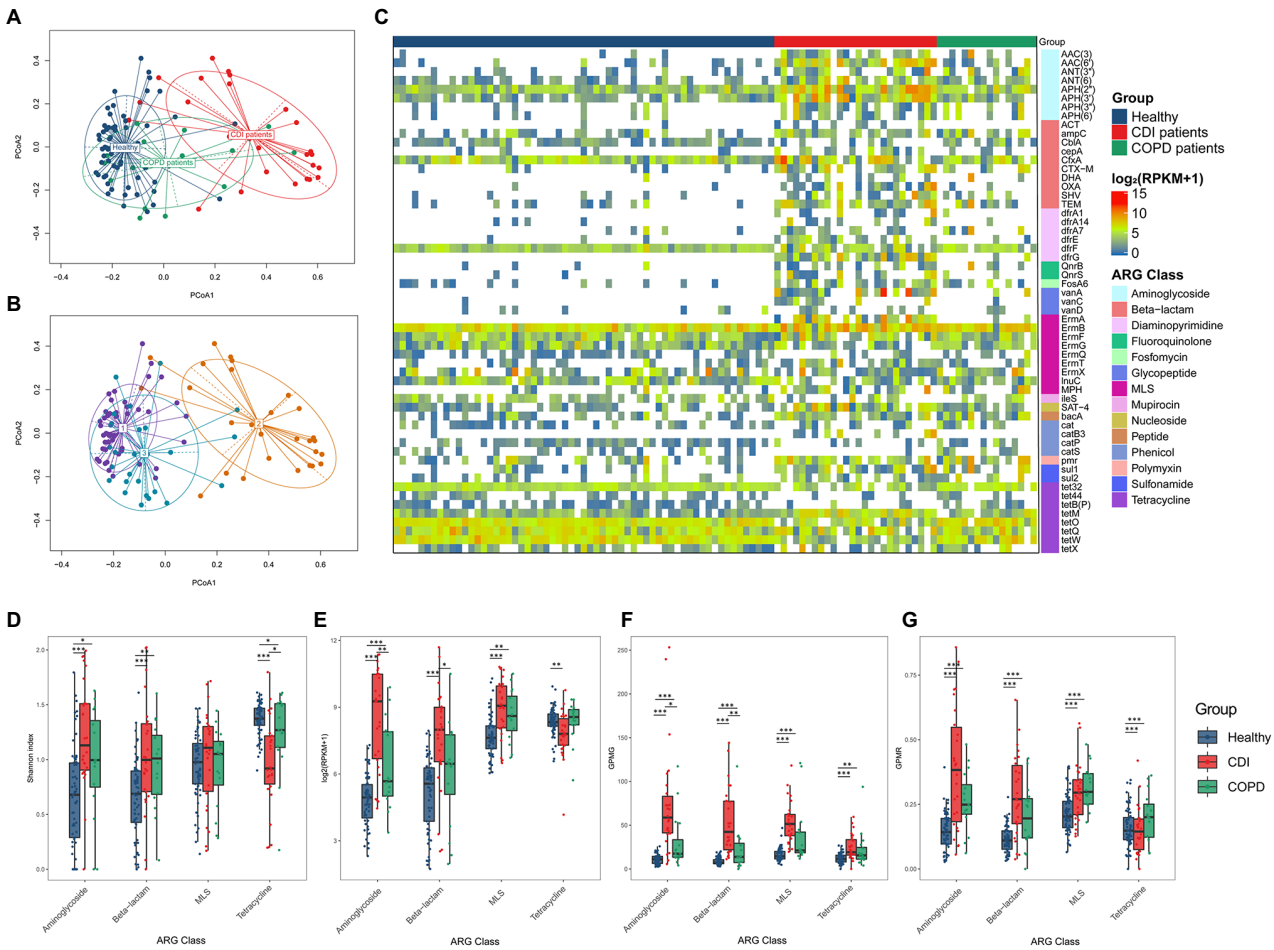
ARG composition in three groups. PCoA result of ARG composition labeled **(A)** by groups, and **(B)** by DMM (k = 3) cluster resulted by bacteria composition. **(C)** Prevalence of ARG family (≥ 30% of prevalence in any group). Heatmap cell represents log_2_ (*RPKM*+1). **(D–G)** Diversity and abundance of four ARG classes were measured using four different metrics: **(D)** Alpha diversity of ARGs, **(E)** ARG abundance in the number of reads per kilobase per million mapped reads (RPKM), **(F)** Gene counts per million genes (GPMG), and **(G)** Gene counts per million reads (GPMR).

The composition of antibiotic resistance genes (ARGs) was distinct among the CDI, COPD, and healthy groups (Figure 2). We identified individual ARGs and placed them in the ARG families according to the ARG ontology in CARD (McArthur et al., 2013). For example, the *bla*_TEM_ genes (e.g., *bla*_TEM-1_ and *bla*_TEM-2_) were identified and grouped under the ARG family name TEM. In the embedding of samples based on the abundance of the ARG families, healthy individuals were tightly clustered together, while the CDI samples were dispersed (Figure 2A). The COPD patients were placed between the CDI and healthy samples. Notably, the CDI samples had the most diverse ARG families (24, 30, and 38.5 ARG families (median) in healthy, COPD, and CDI groups, respectively) as reflected in the PCoA plot (Figure 2A). When the PCoA with ARG families was labeled by the DMM clusters resulting from the bacterial composition, a strong association was observed between the samples grouped using bacteria composition and ARG proportion (Figures 2A,B). When the clusters resulting from the bacterial and ARG compositions were compared, 66% of the samples fell into the same cluster (Supplementary Figure S3).

We found 57 ARG families prevalent in at least one group (≥ 30% of the population; Figure 2C; Supplementary Table S5). Among those 57 ARG families, 28 were prevalent in the CDI group and 13 in the COPD group but were scarce in healthy individuals. All genes prevalent in the COPD group were also prevalent in the CDI group. In addition, 29 genes prevalent in healthy individuals were also prevalent in the CDI or the COPD group.

When the abundance of ARG families was compared among the three groups, the median RPKM for all ARGs in the healthy, COPD, and CDI groups were 684.2, 1215.2, and 2025.0, respectively. Among the 57 prevalent ARG families, six showed significantly different abundance distribution among all three groups (*p* < 0.05): AAC (6′), AAC (3), APH (3′), *bla*_SHV_, tet32, and bacA (Table 2; Figure 2C). Compared to the healthy group, the average abundance of 34 ARG families was higher in the CDI group (*p* < 0.05, CDI vs. healthy) and 26 in the COPD group (*p* < 0.05, COPD vs. healthy; Supplementary Table S5). For example, APH (2″) was found in most samples (100, 93.75, and 100% in healthy, COPD, and CDI, respectively; Supplementary Table S5), but showed significantly different abundances between the groups (*p* < 0.05 for all pairs; 23.6, 45.7, and 334.6 RPKM for average abundance in the healthy, COPD, and CDI groups, respectively). In contrast, the prevalence and abundance of tetQ in all three groups were comparable (prevalence: 100, 93.8, and 80.77%; RPKM for average abundance: 84.9, 54.9, and 87.5 in the healthy, COPD, and CDI groups, respectively).

**Table 2:** ARG families of differential abundance in the three groups.

ARG family^+^	Prevalence			Abundance (mean/median)		
	Healthy	CDI	COPD	Healthy	CDI	COPD
AAC(6′)	21.31	92.31	50.00	1.3/0.0	146.34/73.98	11.43/0.27
AAC(3)	6.56	69.23	31.25	0.15/0.0	13.37/3.74	39.41/0.0
APH(3″)	13.11	69.23	31.25	1.36/0.0	18.20/0.96	22.39/0.0
tet32	100.00	65.38	87.50	37.02/36.37	16.65/8.27	44.18/47.97
SHV	9.84	57.69	31.25	0.20/0.0	37.35/0.63	4.41/0.0
bacA	40.98	80.77	81.25	4.37/0.0	20.54/7.6	34.72/3.84

+ Among the 57 prevalent ARG families, those with statistical significance among the three groups (p < 0.05) were selected. Value of p by Kruskal-Wallis test.

We further investigated the diversity and abundance of ARGs using four different metrics: the Shannon index, RPKM, GPMG, and GPMR (Figures 2D–G). For comparison, we chose the four most prevalent ARG classes—aminoglycosides, β-lactams, MLS, and tetracycline. In general, the tetracycline class showed a different ARG diversity pattern than aminoglycoside, β-lactam, and MLS (Figure 2D). For these three, healthy individuals had the lowest alpha diversity (i.e., Shannon index), followed by the COPD and CDI groups. CDI showed the lowest diversity for tetracycline, followed by the COPD and healthy groups. The healthy and COPD groups had higher median RPKM values of tetracycline abundance than the CDI group. In contrast, the abundance of the other three classes was higher in the CDI group than in the other two groups (Figure 2E). The CDI group showed a significantly higher ARG ratio to the total number of genes predicted in the microbiome (i.e., GPMG) for all four classes since it harbored fewer genes in the gut microbiome (Figure 2F). This was due to the dominance of *Enterococcus* in the CDI group. The median GPMGs for the healthy, COPD, and CDI groups were 64.1, 102.5, and 299.8.

### Correlation Among Bacterial Composition, ARGs, and Medical Histories

**Figure 3:**
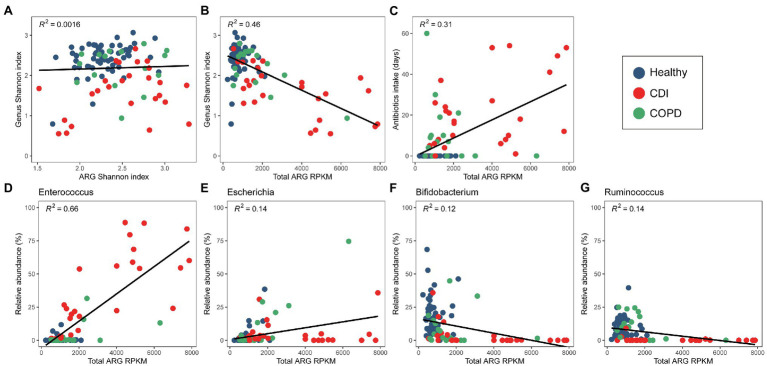
Correlation between bacterial and ARG diversity and abundance. **(A)** Diversity of bacterial and ARG composition. **(B)** Diversity of bacterial composition and ARG abundance. **(C)** Antibiotic intake period and ARG abundance. **(D–F)** ARG abundance and relative abundance of **(D)** *Enterococcus*, **(E)** *Escherichia*, and **(F)** *Bifidobacterium*, **(G)** *Ruminococcus.*

We observed an association between the microbial distribution and ARG abundance (Figure 3). Although bacterial diversity varied among the healthy, COPD, and CDI groups (*p* < 0.05 in all group pairs), the ARG alpha diversity did not show a strong correlation with bacterial alpha diversity (Figure 3A). However, we observed a negative correlation between bacterial diversity and total ARG abundance—samples with high ARG abundance showed lower genus diversity (Figure 3B). When the total ARG abundance and genus abundance was compared, the highest positive correlation was seen with *Enterococcus*, the most abundant genus in the CDI group (Figure 3D). *Escherichia* also showed a low-level positive correlation in the COPD samples (Figure 3E). In contrast, *Bifidobacterium* and *Ruminococcus* were negatively correlated with ARG abundance (Figures 3F,G). Correlation between genus and an ARG were also analyzed (Supplementary Figure S4).

When the medical history of antibiotic use was compared with the abundance of a specific genus, we found *Enterococcus* and *Escherichia* to be positively correlated with the days of antibiotic therapy (DOT), admission history, and patient category (Table 3). However, *Ruminococcus*, *Bifidobacterium*, *Faecalibacterium*, and *Eubacterium* showed a negative correlation. The Shannon index of bacterial composition showed a strong correlation with DOT (≤ 60 days), admission history, and patient category (rho = −0.444, *p* < 0.001; rho = −0.348, *p* < 0.001; rho = −0.56, *p* < 0.001, respectively). The Shannon index of ARGs was positively correlated with DOT (≤ 60 days) and the patient category (rho = 0.282, *p* < 0.01; rho = 0.29, *p* < 0.01, respectively). The abundance of ARGs was positively correlated with DOT (≤ 60 days), admission history, patient category, and history of COPD (rho = 0.524, *p* < 0.001; rho = 0.304, *p* < 0.001; rho = 0.573, *p* < 0.001; rho = 0.464, *p* < 0.001, respectively). The CDI samples showed higher ARG abundance and longer periods of antibiotic intake (Figure 3C). However, the period of antibiotic intake was limited to 60 days before sampling and might not represent the total antibiotic consumption by each person. Nevertheless, it can infer a pattern of antibiotic intake in a short period among the groups.

**Table 3:** Correlation between clinical characteristics and bacterial or antibiotic resistance genes (ARG) distribution.

		DOT of antibiotics (< 60 days)		Category		Admission history		COPD	
		rho	*Value of p*	rho	*Value of p*	rho	*Value of p*	rho	*Value of p*
Bacteria	Shannon index	−0.444	<0.001	−0.56	<0.001	−0.348	<0.001	−0.131	0.186
	*Enterococcus*	0.66	<0.001	0.759	<0.001	0.503	<0.001	0.135	0.175
	*Escherichia*	0.295	0.002	0.401	<0.001	0.218	0.027	0.29	0.003
	*Ruminococcus*	−0.424	<0.001	−0.559	<0.001	−0.475	<0.001	0.044	0.661
	*Bifdobacterium*	−0.631	<0.001	−0.583	<0.001	−0.34	<0.001	−0.168	0.09
	*Faecalibacterium*	−0.605	<0.001	−0.725	<0.001	−0.452	<0.001	−0.244	0.013
	*Eubacterium*	−0.522	<0.001	−0.626	<0.001	−0.409	<0.001	−0.075	0.454
ARG	Abundance (RPKM)	0.524	<0.001	0.573	<0.001	0.304	0.002	0.464	<0.001
	Shannon index	0.282	0.004	0.29	0.003	0.17	0.086	0.12	0.227
	SHV	0.486	<0.001	0.482	<0.001	0.305	0.002	0.051	0.609

DOT, days of therapy; category, category of healthy people, chronic obstructive pulmonary diseases, and Clostridioides difficile infections; COPD, chronic obstructive pulmonary disease; RPKM, reads per kilobases per million reads; SHV, SHV β-lactamases.

### Mobilome Patterns of the Prevalent Aminoglycoside-Resistant Gene AAC(6′)-Ie-APH(2″)-Ia

To find whether the transferring carriers of ARGs are the same among the groups or not, we performed genome-level analysis on mobilomes and neighboring genes of ARGs. The most prevalent ARG in the aminoglycoside class was AAC(6′)-Ie-APH(2″)-Ia, found in more than 99% of the samples (61, 15, and 26 samples in the healthy, COPD, and CDI groups, respectively; Supplementary Table S5). This fused gene has been observed with nearby transposases in both chromosomes and plasmids in a limited number of bacterial species such as *Enterococcus faecium, Enterococcus faecalis, Lactobacillus salivarius,* and *Staphylococcus aureus* (Rouch et al., 1987; Tenorio et al., 2001), and showed high-level gentamicin resistance (Patterson and Zervos, 1990). A previous study reported that *E. faecalis* isolates harboring AAC(6′)-Ie-APH(2″)-Ia tends to be resistant to multiple antibiotics (Costa et al., 2019). In our analysis, *Enterococcus* exhibited a high-level correlation with the abundance of AAC(6′)-Ie-APH(2″)-Ia with statistical significance (*value of p* < 0.001 and rho > 0.7; Supplementary Table S6).

**Figure 4:**
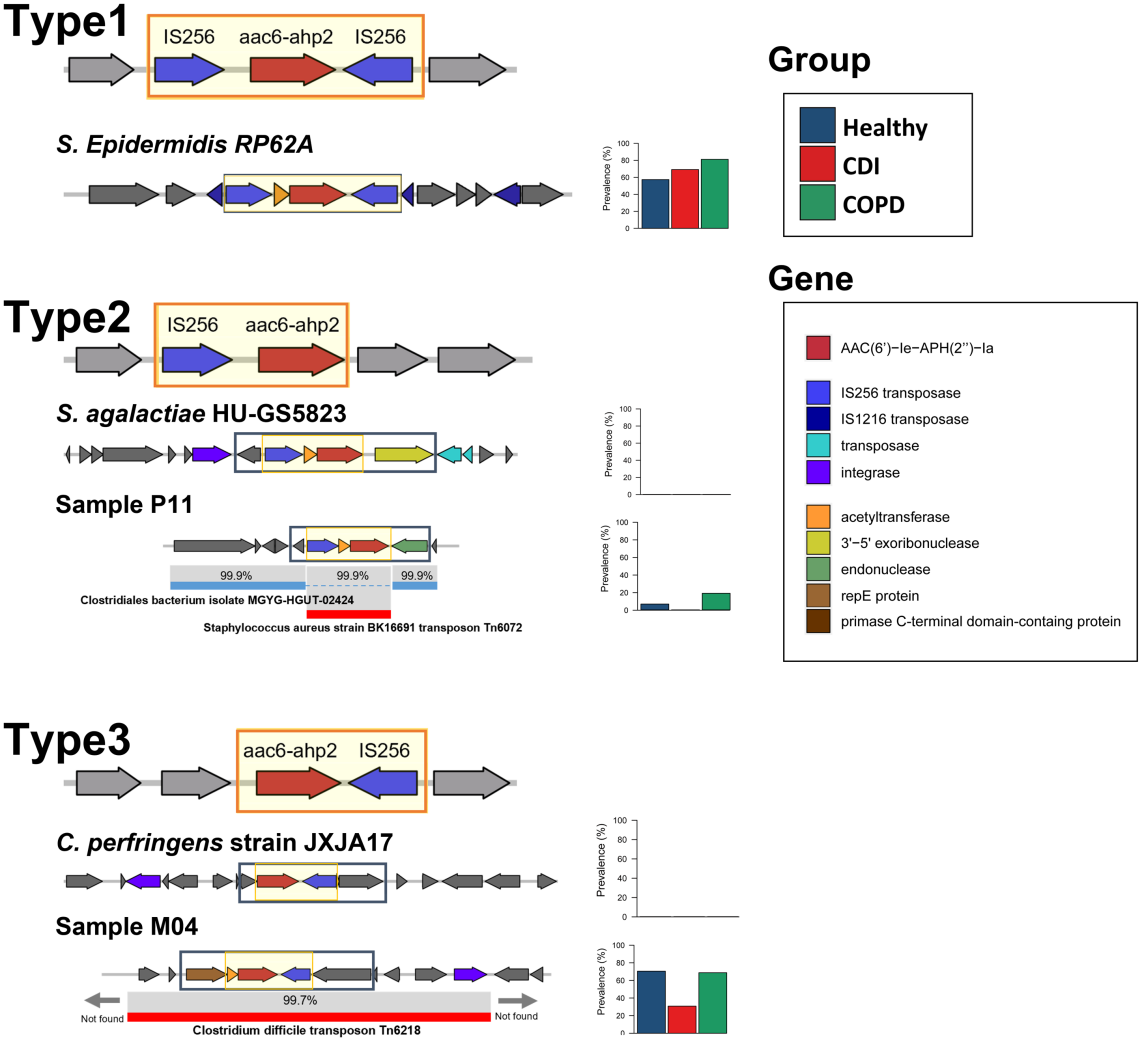
The mobilome patterns and prevalence of AAC(6′)-Ie-APH(2″)-Ia. The backbone sequence is in the black rectangle. The prevalence is plotted in the stacked bar.

We have found three types of mobilome structures according to the position of IS256 transposases after analyzing five neighboring genes of AAC(6′)-Ie-APH(2″)-Ia in the assembled contigs (Figure 4). In addition, the prevalence of each type was measured to determine the dominant mobilome structure in the population. The first structure, which has IS256 transposases on both sides of AAC(6′)-Ie-APH(2″)-Ia (type 1 in Figure 4), was identified as the most prevalent type in the samples. In particular, the AAC(6′)-Ie-APH(2″)-Ia mobilome structure observed in Tn4001 was found in 67 samples (59.0, 86.7, and 69.2% of AAC(6′)-Ie-APH(2″)-Ia containing samples in the healthy, COPD, and CDI groups, respectively). Although this mobilome was highly prevalent, its bacterial origin could not be identified because of the short length of the assembled contigs.

The second structure, which has IS256 transposase only on the same strand of AAC(6′)-Ie-APH(2″)-Ia (type 2 in Figure 4), had a low prevalence. This structure was also scarce in most annotated bacterial genomes in public repositories. A type 2-containing contig was partially aligned with two different species: AAC(6′)-Ie-APH(2″)-Ia and IS256 transposase region were aligned to *S. aureus* transposon Tn6072 with 99.9% similarity and the other regions of the contig to *Clostridiales bacterium* with 99.9% similarity. This finding suggests that the ARG mobilome from Tn6072 was inserted into the *Clostridiales bacterium.* On the other hand, the third structure, which has the IS256 transposase only on the reverse strand of AAC(6′)-Ie-APH(2″)-Ia (type 3 in Figure 4), was also highly prevalent in the samples. When compared with the annotated transposons, this structure along with its neighboring genes, was homologous (> 99%) to that of *C. difficile* Tn6218 first reported in China (Zhao et al., 2020).

The metagenomic approach in this study enabled the discovery of the mobilome structures and their prevalence and identified the dominant carriers of AAC(6′)-Ie-APH(2″)-Ia in the population. Without confining to a specific strain or species, our method found that all subjects except one harbored AAC(6′)-Ie-APH(2″)-Ia genes with the dominant mobilome structures being IS256 transposases on both sides and in the reverse strand. A similar pattern was observed in a previous PCR-based study limited to enterococcal strains in Japan (Watanabe et al., 2009).

### Mobilome Patterns of Prevalent β-Lactamamases

**Figure 5:**
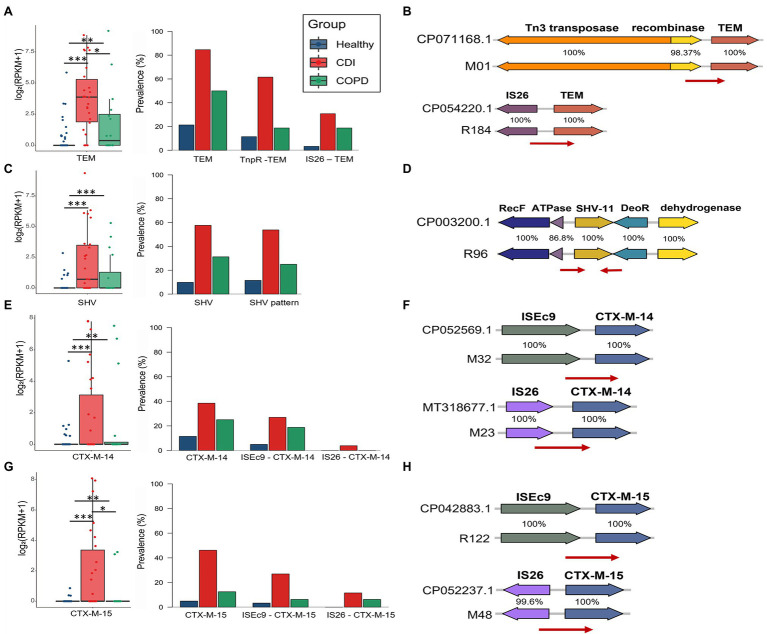
Abundance and prevalence of ARGs and their mobilome. ARG abundance was measured using *log*_2_ (*RPKM*+1) in the boxplot, visualizing its prevalence with stack bars. **(A,B)** TEM, **(C,D)** SHV, **(E,F)** CTX-M-14, and **(G,H)** CTX-M-15.

Among the β-lactamases, *bla*_TEM_, *bla*_SHV_, and *bla*_CTX-M_ were the most prevalent in this study. We used the backbone sequences of the ARG mobilome obtained by searching the assembled contigs and the reference genomes. We estimated the prevalence of specific mobilome structures for the β-lactamase genes. Overall, each mobilome pattern was observed similarly to the ARG proportion in CDI patients, COPD patients, and healthy individuals. In total, 41.7% of all samples contain *bla*_TEM_ genes (21.3, 50, and 84.6%, in healthy, COPD, and CDI samples, respectively) and *bla*_TEM-1_ was the most prevalent in the samples—60.5% of which was carried by Tn1 (11.5, 18.8, and 61.5%, in healthy, COPD, and CDI groups, respectively; Figure 5A). The minor type of mobilome for *bla*_TEM-1_ contained IS26 transposase, found in 30.2% of *bla*_TEM-1_-carrying samples. In the case of *bla*_SHV_, a dominant gene arrangement was observed, which constitutes 96.2% of the *bla*_SHV_-carrying samples and comprises RecF, ATPase, *bla*_SHV_, DeoR, and ygbJ without any nearby transposase (Figure 5D). This highly conserved structure of chromosomal *bla*_SHV_ has also been observed with nearby IS26 transposase for the mobile *bla*_SHV_ (Ford and Avison, 2004). Notably, *bla*_CTX-M-14_ and *bla*_CTX-M-15_—the most prevalent extended-spectrum β-lactamases (ESBLs) worldwide—showed similar mobilome patterns (Figures 5F,H). The most dominant mobilome contained ISEc9 transposase in our samples (61.9 and 58.8% of the samples with *bla*_CTX-M-14_ and *bla*_CTX-M-15_, respectively). The minor mobilome had IS26 transposase, found in 4.8 and 23.5% of the samples that contained *bla*_CTX-M-14_ and *bla*_CTX-M-15_, respectively.

## Discussion

In this study, we compared the bacterial composition of the gut in three groups of people (healthy individuals and COPD and CDI patients) and determined its association with antibiotic use and ARGs. These three groups have experienced different patterns of antibiotic treatment. While healthy individuals had minimum exposure to antibiotics, patients with COPD represent the group with chronic intermittent antibiotic use, and patients with CDI were currently receiving antibiotics. Within the last 60 days before stool collection, COPD patients were antibiotic-free for a median of 40 days and CDI patients were antibiotic-free for 0 days, whereas all healthy individuals were antibiotic-free for 60 days. Since it is difficult to obtain a lifetime history of antibiotic treatment for the subjects, we chose two different disease groups that had different patterns of antibiotic treatment and recorded the recent treatments. The COPD patients generally used β-lactam or fluoroquinolones for <14 days, except for one patient who received macrolide for a month. In contrast, the CDI group received more various broad-spectrum antibiotics such as carbapenem, β-lactam/β-lactam inhibitors, vancomycin, and fluoroquinolones. Thus, our data implicate the impact of the type, amount, and duration of antibiotic administration on the human gut microbiome.

We enrolled COPD patients with chronic bronchitis and emphysema with different exacerbation frequencies from an outpatient clinic. Two-thirds of them had received antibiotics during the last 2 months before sampling. Despite their history of antibiotic use, microbial diversity was relatively well conserved. However, it was slightly lesser than that in healthy individuals (median Shannon index, 2.4 vs. 2.5), and the bacterial composition showed little difference. The *Ruminococcus* genus represented a major portion of the gut microbiome together with *Lactobacillus* and *Escherichia* genera in the COPD patients. In a recent study, *Streptococcus, Rotia*, and *Lachnospiraceae* genera were reported as the dominant genera in the gut microbiome of stable COPD patients (Bowerman et al., 2020). These three genera were not the dominant ones in our study, but we observed a strong influence of antibiotic use on the bacterial composition.

The CDI patients were on broad-spectrum antibiotics at the time of sample collection. The diversity of their gut microbiome was much lesser than the COPD and healthy groups. A characteristic finding in the bacterial composition of CDI patients was the dominance of *Enterococcus*, *Lactobacillus, Bacteroides*, and *Escherichia,* and their diversity was markedly decreased. Our previous study categorized patients with CDI into *Enterococcus*-rich and *Bacteroides*-*Lactobacillus*-rich groups. The *Enterococcus*-rich group had lesser diversity and more antibiotic use than the *Bacteroides-Lactobacillus*-rich group (Kim et al., 2020). Together with these findings, we speculated that the *Enterococcus* genus is generally dominant in guts most severely disrupted by antibiotics.

The overall ARG abundance was the highest in the CDI group, as expected. The ARGs against aminoglycosides, β-lactams, and MLSs showed significant differences among the three groups. Macrolide, β-lactam, and quinolone antibiotics are the most commonly prescribed antibiotics worldwide (ECDC, 2017), and resistance genes against aminoglycosides, β-lactams, and MLS were the most abundant, especially in the CDI and COPD groups. Although aminoglycosides have not been commonly used in human medicine, the enhancement of aminoglycoside resistance genes in patients with COPD or CDI might be due to the enrichment of *Enterococcus* and Enterobacteriaceae in the gut of these patients.

Certain ARGs have been reported among the same species in different niches and even among different species. The horizontal transfer has been considered one of the major mechanisms for spreading ARGs. We, thus, estimated the prevalence of specific mobilome structures for highly abundant β-lactamase genes in this study. For each ARG, several types of mobilome were observed with a similar proportion in all three groups. For *bla*_TEM_ and *bla*_CTX-M_, more than half of the *bla* genes exist with a type of mobile element, which is with Tn3 transposase for *bla*_TEM_ and with ISEc9 transposase for *bla*_CTX-M_. For *bla*_SHV_, a type of gene arrangement was observed in its neighbor. Compared to the PCR-based analysis that targets a specific type of mobile element, this study could successfully profile the distribution of mobilome types that were prevalent in the community.

Despite the merits of these data, there are several limitations. As we selected the three groups based on the duration and amount of antibiotic treatment, their age and the number of comorbidities in the groups differed. The CDI group was heterogeneous, and the members had diarrhea caused by *C. difficile* after antibiotic treatment. Meanwhile, the COPD patients uniformly had lung disease with chronic obstruction, although the lung pathology differed, and most of them had variable comorbidities.

In summary, we identified many ARGs in antibiotic-affected gut microbiomes and correlated the abundance of resistomes, antibiotic use, and gut bacterial diversity.

## Data Availability Statement

The datasets presented in this study can be found in online repositories. The names of the repository/repositories and accession number(s) can be found at: https://www.ebi.ac.uk/ena, PRJEB46960.

## Ethics Statement

The studies involving human participants were reviewed and approved by the Institutional Review Boards (IRB number: HYUH 2016–05-031 and HYUH 2017–06-001 from Hanyang University Hospital and GURI 2016–05-003 from Hanyang University Guri Hospital). The patients/participants provided their written informed consent to participate in this study.

## Author Contributions

MR and HP designed the study. JK and HP performed data acquisition and analysis. YC and MR performed bioinformatic analysis. All authors wrote, read, and approved the final manuscript.

## Funding

This research was supported by a grant (2017ER540702) from the Research of Korea Centers for Disease Control and Prevention and a grant from the BK21 FOUR (Fostering Outstanding Universities for Research) project of the National Research Foundation of Korea Grant.

## Conflict of Interest

The authors declare that the research was conducted in the absence of any commercial or financial relationships that could be construed as a potential conflict of interest.

## Publisher’s Note

All claims expressed in this article are solely those of the authors and do not necessarily represent those of their affiliated organizations, or those of the publisher, the editors and the reviewers. Any product that may be evaluated in this article, or claim that may be made by its manufacturer, is not guaranteed or endorsed by the publisher.

## Supplementary Material

Supplementary Figure S1 Bacterial composition at the genus level.

Supplementary Figure S2 Bacterial composition in three clusters.

Supplementary Figure S3 Cluster assignments based on bacterial composition and ARGs.

Supplementary Figure S4 Correlation between bacterial and ARG abundance.
