# Supplementary figures and images for: Deciphering Resistome in Patients With Chronic Obstructive Pulmonary Diseases and Clostridioides difficile Infections
Source: Front Microbiol. 2022 Aug 2;13:919907. doi: 10.3389/fmicb.2022.919907 (PMC9378971; doi:10.3389/fmicb.2022.919907)

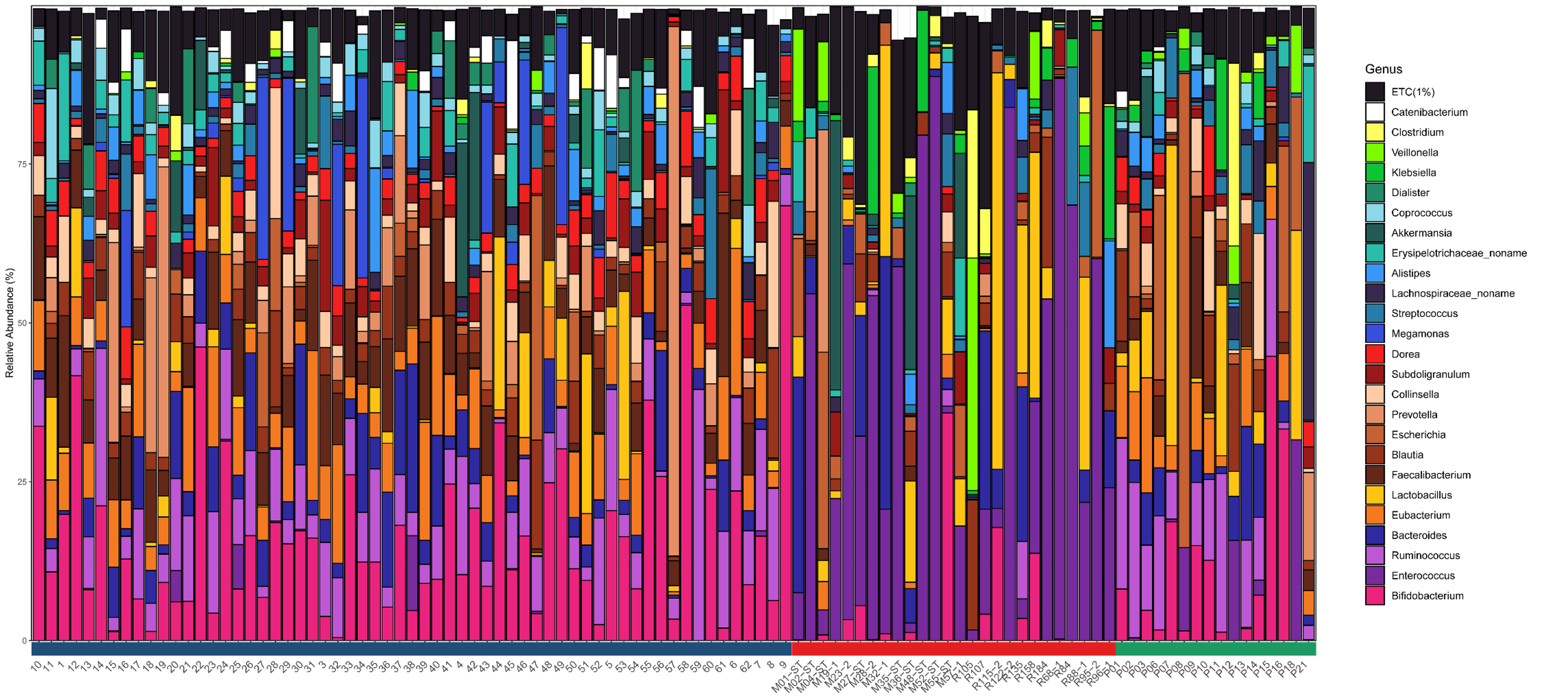

Supplement: Supplementary Figure S1 — Bacterial composition at the genus level. [file Image_1.TIF]

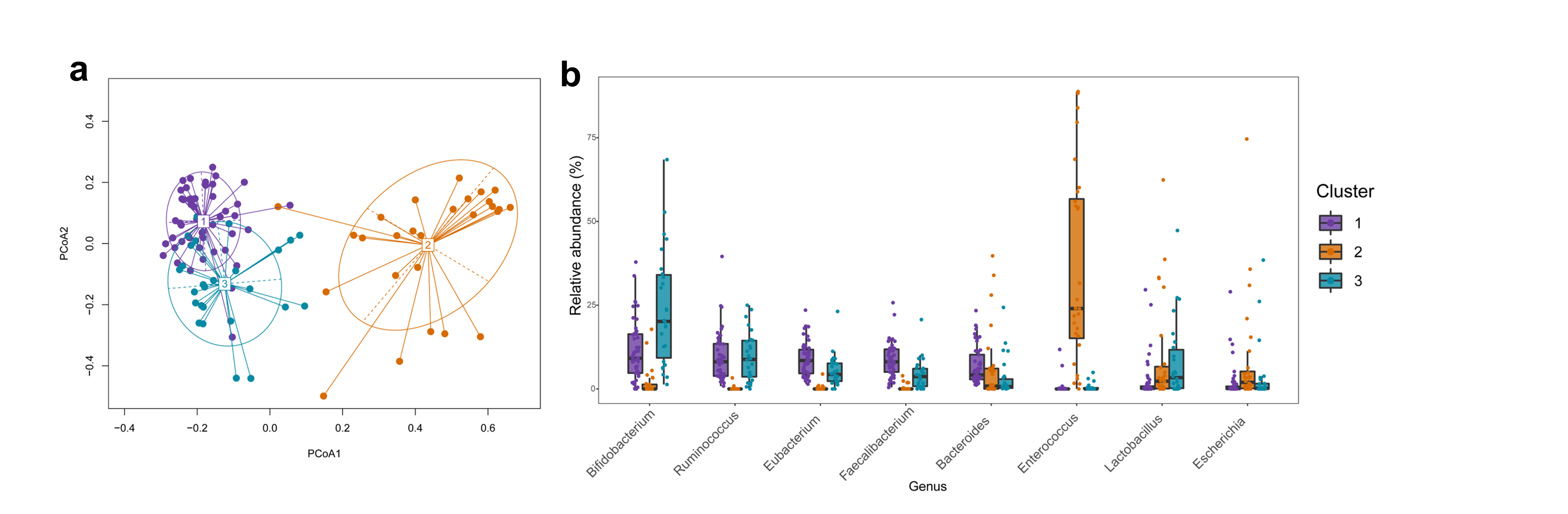

Supplement: Supplementary Figure S2 — Bacterial composition in three clusters. [file Image_2.TIF]

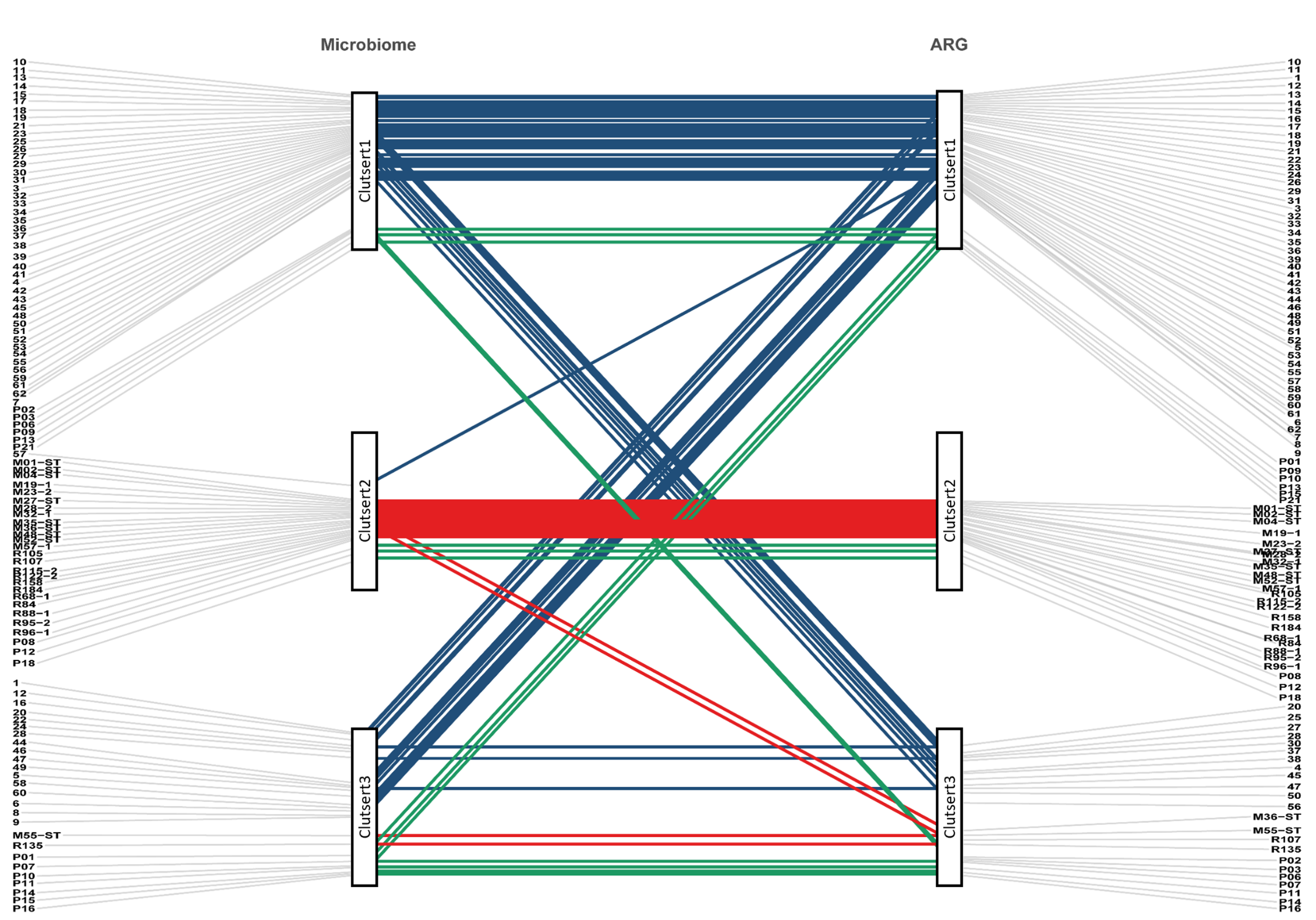

Supplement: Supplementary Figure S3 — Cluster assignments based on bacterial composition and ARGs. [file Image_3.TIF]

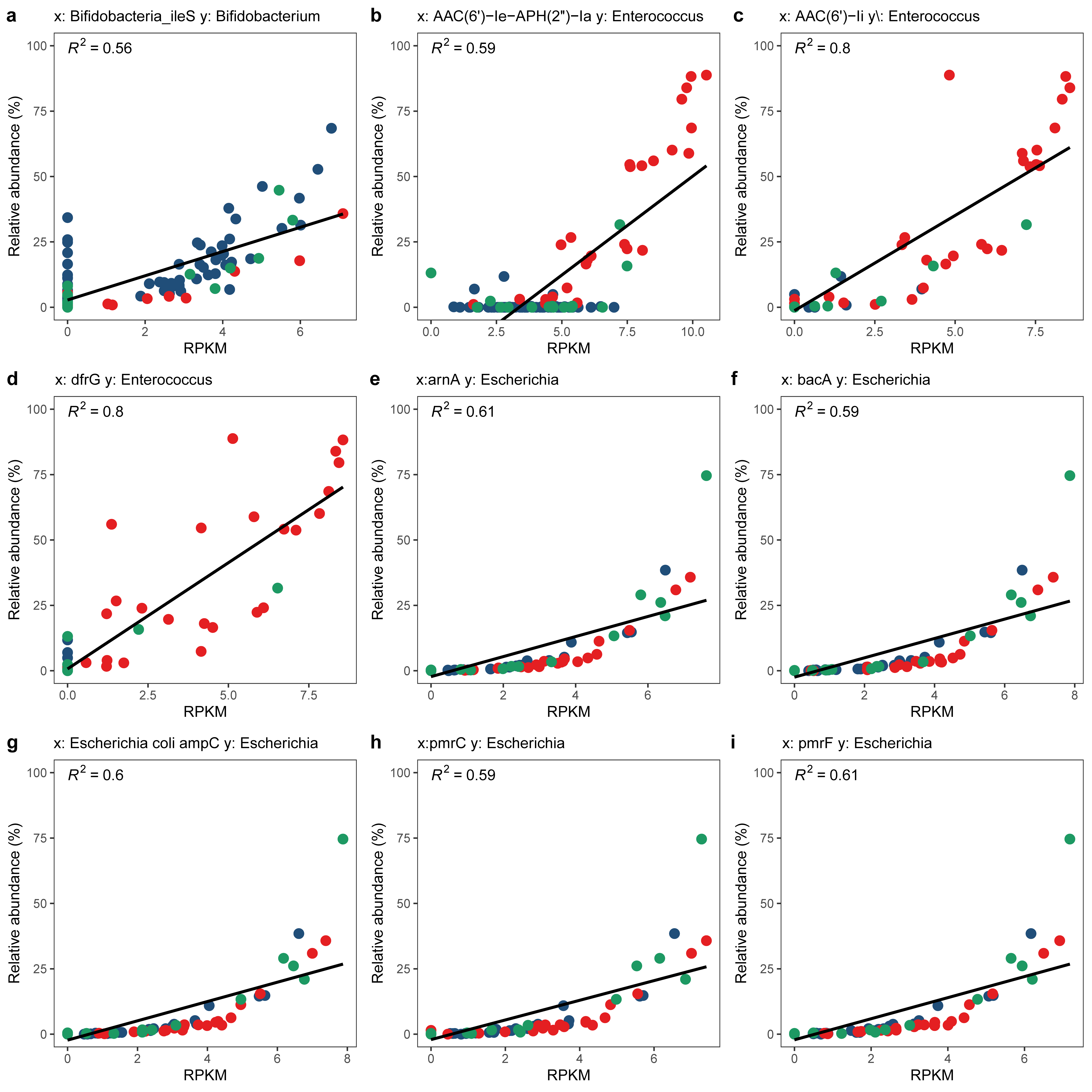

Supplement: Supplementary Figure S4 — Correlation between bacterial and ARG abundance. [file Image_4.TIF]
