# Supplementary material for: Deciphering Resistome in Patients With Chronic Obstructive Pulmonary Diseases and Clostridioides difficile Infections
Source: Front Microbiol. 2022 Aug 2;13:919907. doi: 10.3389/fmicb.2022.919907 (PMC9378971; doi:10.3389/fmicb.2022.919907)
Supplement: Supplementary file 5 [file Table_1.DOCX]

Table S1. List of Charlson's comorbidity scores

|  |  | Healthy (N=61) | COPD (N=16) | CDI (N=26) | p-value |
| --- | --- | --- | --- | --- | --- |
| Charlson's comorbidity score | median  (1Q, 3Q) | 0 (0, 0) | 3 (2.3, 3) | 3 (1, 5) | <0.001* |
| Myocardial infarction | n (%) | 0 | 2 (12.5) | 0 | 0.572 |
| Congestive heart failure | n (%) | 0 | 2 (12.5) | 2 (7.7) | 0.046 |
| Peripheral vascular disease | n (%) | 0 | 1 (6.3) | 2 (7.7) | 0.039 |
| Cerebrovascular disease | n (%) | 0 | 2 (12.5) | 8 (30.8) | <0.001 |
| Dementia | n (%) | 0 | 1 (6.3) | 5 (19.2) | 0.001 |
| COPD | n (%) | 0 | 16 (100) | 5 (19.2) | 0.001 |
| Connective tissue disease | n (%) | 0 | 1 (6.3) | 4 (15.4) | 0.002 |
| Peptic ulcer disease | n (%) | 0 | 1 (6.3) | 1 (3.8) | 0.162 |
| Mild liver disease | n (%) | 0 | 1 (6.3) | 0 | 0.691 |
| Diabetes | n (%) | 0 | 7 (43.8) | 5 (19.2) | 0.001 |
| Hemiplegia | n (%) | 0 | 0 | 1 (3.8) | 0.117 |
| Moderate to severe renal disease | n (%) | 0 | 2 (12.5) | 9 (34.6) | <0.001 |
| Diabetes with organ damage | n (%) | 0 | 1 (6.3) | 3 (11.5) | 0.01 |
| Any tumor within last 5years | n (%) | 0 | 5 (31.3) | 2 (7.7) | 0.046 |
| Lymphoma | n (%) | 0 | 0 | 2 (7.7) | 0.026 |
| Leukemia | n (%) | 0 | 0 | 0 |  |
| Moderate to severe liver disease | n (%) | 0 | 1 (6.3) | 1 (3.8) | 0.162 |
| Metastatic solid tumor | n (%) | 0 | 0 | 2 (7.7) | 0.026 |
| AIDS | n (%) | 0 | 0 | 0 |  |

Table S2. Antibiotics treatment history of CDI patients.

| patient  number | Total  (DOT/DDD) ^1^ | vancomycin | Cephalosporin  (1st&2nd)^2^ | Broad  cephalosporin^3^ | carbapenem | quinolone | metronidazole | tetracycline | BL/BLI^4^ | colistin | amikacin | macrolide | clindamycin |
| --- | --- | --- | --- | --- | --- | --- | --- | --- | --- | --- | --- | --- | --- |
| 1 | 16/8.5 | 10/3.5 |  | 4/4 |  |  |  |  |  |  |  |  | 2/1 |
| 2 | 49/49 |  |  |  | 49/49 |  |  |  |  |  |  |  |  |
| 3 | 24/25 |  |  | 12/14 |  | 12/11 |  |  |  |  |  |  |  |
| 4 | 77/103.5 | 10/10 | 7/4 |  | 21/21 |  | 10/7.5 | 29/58 |  |  |  |  |  |
| 5 | 68/77.3 |  |  | 13/19.5 | 2/1.7 | 12/15.5 | 3/3 | 3/3 | 11/10.6 | 24/24 |  |  |  |
| 6 | 8/5.3 |  | 8/5.3 |  |  |  |  |  |  |  |  |  |  |
| 7 | 2/1.3 |  |  |  |  |  |  |  | 2/1.3 |  |  |  |  |
| 8 | 28/27.8 |  |  | 4/4 | 14/14 |  | 4/4 |  | 6/5.8 |  |  |  |  |
| 9 | 12/12 |  |  |  | 10/10 |  | 2/2 |  |  |  |  |  |  |
| 10 | 0/0 |  |  |  |  |  |  |  |  |  |  |  |  |
| 11 | 10/10 |  | 4/4 | 5/5 |  | 1/1 |  |  |  |  |  |  |  |
| 12 | 8/12 |  |  |  |  | 8/12 |  |  |  |  |  |  |  |
| 13 | 5/1.3 |  |  | 5/1.3 |  |  |  |  |  |  |  |  |  |
| 14 | 17/8.4 | 2/1 |  |  | 12/6 |  |  |  | 3/1.4 |  |  |  |  |
| 15 | 19/22.7 |  | 6/6 |  |  |  |  |  | 13/16.7 |  |  |  |  |
| 16 | 53/37.9 | 10/8.2 |  | 2/1.5 | 25/10.3 |  |  |  | 16/17.9 |  |  |  |  |
| 17 | 21/7.1 |  |  | 2/1 |  |  |  |  | 19/6.1 |  |  |  |  |
| 18 | 116/135.1 |  |  | 41/53.5 |  | 18/16 |  |  | 20/22.8 |  | 25/18.8 | 12/24 |  |
| 19 | 62/71.3 | 6/3.6 |  | 14/14 | 5/5 |  | 6/6 |  | 31/42.7 |  |  |  |  |
| 20 | 40/44.7 |  |  | 25/25 |  |  | 14/18.7 |  | 1/1 |  |  |  |  |
| 21 | 4/0.7 |  |  |  | 4/0.7 |  |  |  |  |  |  |  |  |
| 22 | 0/0 |  |  |  |  |  |  |  |  |  |  |  |  |
| 23 | 19/19 | 5/5 | 7/7 | 7/7 |  |  |  |  |  |  |  |  |  |
| 24 | 8/7 |  |  | 8/7 |  |  |  |  |  |  |  |  |  |
| 25 | 3947.8 |  |  | 6/9 |  |  |  |  | 33/38.8 |  |  |  |  |
| 26 | 26/21.5 |  |  | 9/4.5 |  | 17/17 |  |  |  |  |  |  |  |
| No. of use | 24/26 (92.3%) | 6/26 (23.1%) | 5/26 (19.2%) | 15/26 (57.7%) | 9/26 (34.6) | 6/26 (23.1%) | 6/26 (23.1%) | 2/26 (7.7%) | 11/26 (42.3%) | 1/26 (3.8%) | 1/26 (3.8%) | 1/26 (3.8%) | 1/26 (3.8%) |
| total DOT/DDD | 731/756.2 | 43/31.3 | 32/29.3 | 157/170.3 | 142/117.7 | 68/72.5 | 39/41.2 | 32/61 | 155/165.1 | 24/24 | 25/18.8 | 12/24 | 2/1 |
| average DOT/patient | 28.1/29.1 | 1.7/1.2 | 1.2/1.1 | 6.0/6.6 | 5.5/4.5 | 2.6/2.8 | 1.5/1.6 | 1.2/2.3 | 6.0/6.4 | 0.9/0.9 | 1.0/0.7 | 0.5/0.9 | 0.1/0.0 |

Each cell indicates DOT/DDD.

1. DOT days of treatment; DDD defined daily dose, 2. 1st and 2nd generation of cephalosporins, 3. 3rd and 4th generation of cephalosporins, 4. Beta-lactam/beta-lactamase inhibitors, 5. Trimethoprim-sulfamethoxazole

Table S3. Antibiotics treatment history of COPD patients

| COPD | 1year |  |  |  |  |  | 60days |  |  |  |  |
| --- | --- | --- | --- | --- | --- | --- | --- | --- | --- | --- | --- |
| patient number | total | broad cephalosporins | macrolide | quinolone | beta-lactam | septrin | total | broad cephalosporins | macrolide | quinolone | beta-lactam |
| 1 | 309/309 | 9/9 | 300/300 |  |  |  | 40/40 | 9/9 | 31/31 |  |  |
| 2 | 50/50 |  |  | 50/50 |  |  | 10/10 |  |  | 10/10 |  |
| 3 | 39/53.5 | 14/21 |  | 25/32.5 |  |  | 10/10 |  |  | 10/10 |  |
| 4 | 30/37.7 | 7/5.3 |  | 13/19.5 | 10/12.9 |  | 7/5.3 | 7/5.3 |  |  |  |
| 5 | 39/43.8 |  |  |  | 39/43.8 |  | 0/0 |  |  |  |  |
| 6 | 31/38.8 |  |  |  | 31/38.8 |  | 0/0 |  |  |  |  |
| 7 | 93/104.5 | 63/63 |  | 30/41.5 |  |  | 9/13.5 |  |  | 9/13.5 |  |
| 8 | 78/98.9 | 12/7.8 |  | 57/79.5 | 9/11.6 |  | 0/0 |  |  |  |  |
| 9 | 238/527 |  |  |  | 148/518 | 90/9 | 60/240 |  |  |  | 60/240 |
| 10 | 130/178.8 | 12/10.8 |  | 107/157 | 11/11 |  | 21/28 | 7/7 |  | 14/21 |  |
| 11 | 50/47.8 | 31/19.3 |  | 19/28.5 |  |  | 19/17.2 | 14/9.7 |  | 5/7.5 |  |
| 12 | 46/61.3 | 7/5.3 |  | 29/43.5 | 10/12.5 |  | 14/15.8 | 7/5.3 |  | 7/10.5 |  |
| 13 | 55/20 | 5/3.8 |  | 8/12 |  | 42/4.2 | 0/0 |  |  |  |  |
| 14 | 50/50 |  |  | 50/50 |  |  | 0/0 |  |  |  |  |
| 15 | 23/32 | 9/8 | 6/12 | 8/12 |  |  | 0/0 |  |  |  |  |
| 16 | 28/14 | 28/14 |  |  |  |  | 4/2 | 4/2 |  |  |  |
| No. of use | 16/16 (100%) | 11/16 (68.8%) | 2/16 (12.5%) | 11/16 (68.8%) | 7/16 (43.8%) | 2/16 (12.5%) | 10/16 (62.5%) | 6/16 (37.5%) | 1/16 (6.3%) | 6/16 (37.5%) | 1/16 (6.3%) |
| total DOT/DDD | 1289/1667.1 | 197/167.3 | 306/312 | 396/526 | 258/648.6 | 132/13.2 | 194/381.8 | 48/38.3 | 31/31 | 55/72.5 | 60/240 |
| average DOT/DDD per patient | 80.6/104.2 | 12.3/10.5 | 19.1/19.5 | 24.8/32.9 | 16.1/40.5 | 8.3/0.8 | 12.1/23.9 | 3.0/2.4 | 1.9/1.9 | 3.4/4.5 | 3.8/15 |

Each cell indicates DOT/DDD.

Table S4. Bacterial composition in three groups (median values in each group)

| Genus | **Median proportion** | | | | **Average proportion** | | | |
| --- | --- | --- | --- | --- | --- | --- | --- | --- |
|  | **All samples** | **Healthy** | **CDI** | **COPD** | **All samples** | **Healthy** | **CDI** | **COPD** |
| Bifidobacterium | 8.1418 | 12.3645 | 0.0729 | 3.3166 | 11.8797 | 16.1461 | 3.3705 | 9.4412 |
| Enterococcus | 0.0000 | 0.0000 | 23.9883 | 0.0000 | 10.0161 | 0.4337 | 36.2032 | 3.9952 |
| Ruminococcus | 4.4356 | 7.8254 | 0.0000 | 10.0856 | 7.0271 | 8.7082 | 0.4866 | 11.2460 |
| Bacteroides | 3.1656 | 3.3053 | 1.5211 | 2.4563 | 5.8303 | 5.5323 | 7.5018 | 4.2502 |
| Eubacterium | 4.3822 | 7.3314 | 0.0000 | 5.0667 | 5.7595 | 7.8909 | 0.5063 | 6.1698 |
| Lactobacillus | 0.6434 | 0.2411 | 2.4872 | 2.9134 | 5.6492 | 3.1447 | 9.5198 | 8.9079 |
| Faecalibacterium | 4.0298 | 6.9988 | 0.0000 | 2.5393 | 5.2793 | 7.7266 | 0.2622 | 4.1013 |
| Blautia | 2.9946 | 3.3813 | 0.8448 | 3.0679 | 4.1363 | 4.1989 | 3.3232 | 5.2188 |
| Escherichia | 0.2410 | 0.0000 | 1.9067 | 1.1757 | 4.0315 | 1.7660 | 5.1823 | 10.7982 |
| Prevotella | 0.1410 | 0.5058 | 0.0000 | 0.0000 | 3.9625 | 5.5235 | 1.9743 | 1.2423 |
| Collinsella | 1.9349 | 2.8060 | 0.0000 | 1.6240 | 3.1335 | 4.0731 | 0.2174 | 4.2896 |
| Subdoligranulum | 1.7482 | 2.0888 | 0.0000 | 1.5568 | 2.6134 | 3.4402 | 1.4095 | 1.4171 |
| Dorea | 1.6246 | 2.9066 | 0.0000 | 0.4852 | 2.3933 | 3.4886 | 0.0448 | 2.0337 |
| Megamonas | 0.0000 | 0.0000 | 0.0000 | 0.0000 | 2.1416 | 3.6049 | 0.0000 | 0.0430 |
| Streptococcus | 0.7262 | 0.6720 | 0.5944 | 1.9208 | 2.1219 | 1.7263 | 2.5506 | 2.9337 |
| Lachnospiraceae_genus | 1.0562 | 1.5423 | 0.0000 | 1.3623 | 2.0520 | 1.9052 | 0.6372 | 4.9105 |
| Alistipes | 0.3677 | 0.9173 | 0.0000 | 0.2641 | 1.9426 | 2.2103 | 1.5110 | 1.6238 |
| Erysipelotrichaceae_genus | 0.4070 | 1.0467 | 0.3305 | 0.0000 | 1.9407 | 2.2647 | 1.3929 | 1.5961 |
| Akkermansia | 0.0000 | 0.0000 | 0.0000 | 0.0000 | 1.9214 | 1.5796 | 3.4110 | 0.8040 |
| Coprococcus | 0.8380 | 1.5086 | 0.0000 | 0.7842 | 1.4752 | 2.1625 | 0.0046 | 1.2449 |
| Dialister | 0.0000 | 0.0000 | 0.0000 | 0.0000 | 1.4222 | 2.2220 | 0.1091 | 0.5065 |
| Klebsiella | 0.0000 | 0.0000 | 0.7089 | 0.0000 | 1.2428 | 0.1160 | 3.3738 | 2.0759 |
| Veillonella | 0.0000 | 0.0000 | 0.0000 | 0.4209 | 1.1570 | 0.1873 | 3.1462 | 1.6217 |
| Clostridium | 0.0000 | 0.0000 | 0.2718 | 0.0000 | 1.0916 | 0.4550 | 2.0055 | 2.0335 |
| Catenibacterium | 0.0000 | 0.0000 | 0.0000 | 0.0000 | 1.0465 | 1.7109 | 0.0000 | 0.2144 |
| Parabacteroides | 0.3453 | 0.5531 | 0.0000 | 0.2435 | 0.9971 | 0.6765 | 2.0653 | 0.4835 |
| Roseburia | 0.1764 | 0.4556 | 0.0000 | 0.1609 | 0.7831 | 1.0998 | 0.0240 | 0.8094 |
| Peptostreptococcaceae_genus | 0.2160 | 0.2084 | 0.2110 | 0.3028 | 0.5938 | 0.4457 | 0.5123 | 1.2911 |
| Eggerthella | 0.0000 | 0.0000 | 0.0000 | 0.3435 | 0.5682 | 0.5440 | 0.5847 | 0.6339 |
| Anaerostipes | 0.0000 | 0.1455 | 0.0000 | 0.1339 | 0.4382 | 0.5538 | 0.1594 | 0.4507 |
| Adlercreutzia | 0.0000 | 0.2102 | 0.0000 | 0.0000 | 0.3960 | 0.4960 | 0.0000 | 0.6582 |
| Pediococcus | 0.0000 | 0.0000 | 0.0000 | 0.0000 | 0.3783 | 0.0234 | 1.3259 | 0.1915 |
| Coprobacillus | 0.0000 | 0.0000 | 0.0000 | 0.0000 | 0.3352 | 0.1857 | 0.7938 | 0.1598 |
| Barnesiella | 0.0000 | 0.0000 | 0.0000 | 0.0000 | 0.2480 | 0.3582 | 0.0677 | 0.1211 |
| Enterobacter | 0.0000 | 0.0000 | 0.0000 | 0.0000 | 0.2417 | 0.0389 | 0.8536 | 0.0208 |
| Haemophilus | 0.0000 | 0.0000 | 0.0000 | 0.0000 | 0.2111 | 0.1997 | 0.1650 | 0.3298 |
| Phascolarctobacterium | 0.0000 | 0.0000 | 0.0000 | 0.0000 | 0.2051 | 0.3306 | 0.0000 | 0.0598 |
| Weissella | 0.0000 | 0.0000 | 0.0000 | 0.0000 | 0.2028 | 0.2393 | 0.1646 | 0.1257 |
| Bilophila | 0.0000 | 0.1035 | 0.0000 | 0.0000 | 0.1964 | 0.1920 | 0.2850 | 0.0697 |
| Paraprevotella | 0.0000 | 0.0000 | 0.0000 | 0.0000 | 0.1909 | 0.2819 | 0.0000 | 0.1541 |
| Megasphaera | 0.0000 | 0.0000 | 0.0000 | 0.0000 | 0.1396 | 0.1871 | 0.0988 | 0.0248 |
| Leuconostoc | 0.0000 | 0.0000 | 0.0000 | 0.0000 | 0.1297 | 0.1116 | 0.1849 | 0.1088 |
| Mitsuokella | 0.0000 | 0.0000 | 0.0000 | 0.0000 | 0.1289 | 0.2177 | 0.0000 | 0.0000 |
| Staphylococcus | 0.0000 | 0.0000 | 0.0000 | 0.0000 | 0.1256 | 0.0000 | 0.4975 | 0.0000 |
| Odoribacter | 0.0000 | 0.0000 | 0.0000 | 0.0000 | 0.1149 | 0.1223 | 0.1150 | 0.0865 |
| Citrobacter | 0.0000 | 0.0000 | 0.0000 | 0.0000 | 0.1122 | 0.0175 | 0.3937 | 0.0155 |
| Butyrivibrio | 0.0000 | 0.0000 | 0.0000 | 0.0000 | 0.0982 | 0.1657 | 0.0000 | 0.0000 |
| Clostridiales_genus | 0.0000 | 0.0000 | 0.0000 | 0.0000 | 0.0924 | 0.1148 | 0.0967 | 0.0000 |
| Oscillibacter | 0.0000 | 0.0000 | 0.0000 | 0.0000 | 0.0920 | 0.1240 | 0.0431 | 0.0493 |
| Lactococcus | 0.0000 | 0.0000 | 0.0000 | 0.0000 | 0.0900 | 0.0811 | 0.0908 | 0.1224 |
| Bacteroidales_genus | 0.0000 | 0.0000 | 0.0000 | 0.0000 | 0.0860 | 0.1083 | 0.0000 | 0.1408 |
| Morganella | 0.0000 | 0.0000 | 0.0000 | 0.0000 | 0.0805 | 0.0000 | 0.3189 | 0.0000 |
| Turicibacter | 0.0000 | 0.0000 | 0.0000 | 0.0000 | 0.0714 | 0.0000 | 0.0697 | 0.3463 |
| Peptostreptococcus | 0.0000 | 0.0000 | 0.0000 | 0.0000 | 0.0704 | 0.0000 | 0.2789 | 0.0000 |
| Butyricicoccus | 0.0000 | 0.0000 | 0.0000 | 0.0000 | 0.0572 | 0.0000 | 0.2265 | 0.0000 |
| Actinomyces | 0.0000 | 0.0000 | 0.0000 | 0.0000 | 0.0488 | 0.0278 | 0.1044 | 0.0387 |
| Proteus | 0.0000 | 0.0000 | 0.0000 | 0.0000 | 0.0481 | 0.0026 | 0.1845 | 0.0000 |
| Corynebacterium | 0.0000 | 0.0000 | 0.0000 | 0.0000 | 0.0414 | 0.0000 | 0.1640 | 0.0000 |
| Rothia | 0.0000 | 0.0000 | 0.0000 | 0.0000 | 0.0413 | 0.0121 | 0.0535 | 0.1326 |
| Desulfovibrio | 0.0000 | 0.0000 | 0.0000 | 0.0000 | 0.0379 | 0.0055 | 0.1372 | 0.0000 |
| Porphyromonas | 0.0000 | 0.0000 | 0.0000 | 0.0000 | 0.0375 | 0.0024 | 0.1428 | 0.0000 |
| Candida | 0.0000 | 0.0000 | 0.0000 | 0.0000 | 0.0353 | 0.0000 | 0.1398 | 0.0000 |
| Parvimonas | 0.0000 | 0.0000 | 0.0000 | 0.0000 | 0.0265 | 0.0000 | 0.1050 | 0.0000 |
| Fusobacterium | 0.0000 | 0.0000 | 0.0000 | 0.0000 | 0.0265 | 0.0000 | 0.1048 | 0.0000 |

Table S5. Prevalence and abundance of ARGs in three groups

|  |  | Prevalence | | | Abundance(average) | | | Abundance(median) | | | Statistical test(p-value<0.05) | | |  |
| --- | --- | --- | --- | --- | --- | --- | --- | --- | --- | --- | --- | --- | --- | --- |
| Class | Sample | Healthy | CDI | COPD | Healthy | CDI | COPD | Healthy | CDI | COPD | CDI vs Healthy | COPD vs Healthy | CDI vs  COPD | three groups |
| Aminoglycoside | APH(2'') | 100.0000 | 100.0000 | 93.7500 | 23.6315 | 334.6466 | 45.7463 | 15.1050 | 173.1858 | 35.4533 | 0.0000 | 0.0173 | 0.0081 |  |
| Aminoglycoside | AAC(6') | 21.3115 | 92.3077 | 50.0000 | 1.3040 | 146.3391 | 11.4260 | 0.0000 | 73.9779 | 0.2730 | 0.0000 | 0.0388 | 0.0028 | 0.0297 |
| Aminoglycoside | APH(3') | 77.0492 | 92.3077 | 81.2500 | 5.7790 | 197.9790 | 11.6820 | 3.3605 | 62.3653 | 5.9008 | 0.0000 |  | 0.0087 |  |
| Aminoglycoside | ANT(6) | 54.0984 | 76.9231 | 75.0000 | 2.5252 | 45.9620 | 7.6714 | 0.6650 | 11.2954 | 3.8330 | 0.0011 | 0.0059 |  |  |
| Aminoglycoside | AAC(3) | 6.5574 | 69.2308 | 31.2500 | 0.1491 | 13.3658 | 39.4093 | 0.0000 | 3.7407 | 0.0000 | 0.0000 | 0.0440 |  | 0.0283 |
| Aminoglycoside | APH(3'') | 13.1148 | 69.2308 | 31.2500 | 1.3629 | 18.1957 | 22.3854 | 0.0000 | 0.9621 | 0.0000 | 0.0121 | 0.0032 |  | 0.0368 |
| Aminoglycoside | APH(6) | 14.7541 | 61.5385 | 31.2500 | 1.1888 | 16.1371 | 17.0920 | 0.0000 | 0.9316 | 0.0000 | 0.0142 | 0.0033 |  |  |
| Aminoglycoside | ANT(3'') | 21.3115 | 61.5385 | 50.0000 | 0.6822 | 30.8168 | 34.4352 | 0.0000 | 0.7354 | 0.1586 | 0.0284 | 0.0002 |  |  |
| Beta-lactam | TEM | 21.3115 | 84.6154 | 50.0000 | 1.4355 | 59.8026 | 43.5913 | 0.0000 | 13.6289 | 0.3466 | 0.0001 | 0.0241 |  |  |
| Beta-lactam | CfxA | 98.3607 | 80.7692 | 93.7500 | 38.5826 | 245.1717 | 39.2718 | 25.0896 | 42.2602 | 27.4510 | 0.0051 |  |  |  |
| Beta-lactam | ampC | 39.3443 | 76.9231 | 68.7500 | 4.5195 | 20.4025 | 34.4424 | 0.0000 | 8.0294 | 3.3992 | 0.0071 | 0.0011 |  |  |
| Beta-lactam | CTX-M | 14.7541 | 69.2308 | 37.5000 | 0.7339 | 51.7590 | 21.1542 | 0.0000 | 4.9056 | 0.0000 | 0.0001 | 0.0024 |  |  |
| Beta-lactam | OXA | 3.2787 | 65.3846 | 12.5000 | 0.0980 | 56.4666 | 0.2338 | 0.0000 | 0.4972 | 0.0000 | 0.0155 |  |  |  |
| Beta-lactam | SHV | 9.8361 | 57.6923 | 31.2500 | 0.1976 | 37.3525 | 4.4118 | 0.0000 | 0.6334 | 0.0000 | 0.0255 | 0.0017 |  | 0.0312 |
| Beta-lactam | cepA | 13.1148 | 46.1538 | 25.0000 | 0.3108 | 9.0675 | 0.5463 | 0.0000 | 0.0000 | 0.0000 | 0.0013 |  |  |  |
| Beta-lactam | DHA | 1.6393 | 34.6154 | 12.5000 | 1.4679 | 3.3345 | 38.5151 | 0.0000 | 0.0000 | 0.0000 |  |  |  |  |
| Beta-lactam | ACT | 3.2787 | 30.7692 | 0.0000 | 0.0776 | 1.3580 | 0.0000 | 0.0000 | 0.0000 | 0.0000 | 0.0233 |  |  |  |
| Beta-lactam | CblA | 78.6885 | 26.9231 | 62.5000 | 3.9710 | 1.2148 | 5.2043 | 1.8402 | 0.0000 | 1.0251 | 0.0331 |  |  |  |
| Diaminopyrimidine | dfrG | 0.0000 | 92.3077 | 12.5000 | 0.0000 | 80.7588 | 5.9408 | 0.0000 | 17.4207 | 0.0000 | 0.0000 | 0.0416 | 0.0196 |  |
| Diaminopyrimidine | dfrF | 100.0000 | 76.9231 | 87.5000 | 24.4889 | 32.7571 | 24.9049 | 20.6854 | 11.5353 | 25.7622 |  |  |  |  |
| Diaminopyrimidine | dfrE | 4.9180 | 57.6923 | 12.5000 | 0.1180 | 11.3535 | 0.4574 | 0.0000 | 2.2751 | 0.0000 | 0.0001 |  | 0.0425 |  |
| Diaminopyrimidine | dfrA7 | 8.1967 | 42.3077 | 31.2500 | 1.4223 | 3.4029 | 40.9090 | 0.0000 | 0.0000 | 0.0000 |  | 0.0109 |  |  |
| Diaminopyrimidine | dfrA14 | 6.5574 | 34.6154 | 6.2500 | 0.0691 | 12.3234 | 0.4971 | 0.0000 | 0.0000 | 0.0000 | 0.0203 |  |  |  |
| Diaminopyrimidine | dfrA1 | 0.0000 | 34.6154 | 6.2500 | 0.0000 | 26.7780 | 1.6506 | 0.0000 | 0.0000 | 0.0000 |  |  |  |  |
| Fluoroquinolone | QnrB | 6.5574 | 50.0000 | 25.0000 | 1.1610 | 5.1113 | 27.4625 | 0.0000 | 0.1117 | 0.0000 |  |  |  |  |
| Fluoroquinolone | QnrS | 3.2787 | 46.1538 | 12.5000 | 0.0221 | 29.9310 | 2.5732 | 0.0000 | 0.0000 | 0.0000 | 0.0014 | 0.0119 |  |  |
| Fosfomycin | FosA6 | 21.3115 | 69.2308 | 43.7500 | 0.4442 | 13.2249 | 6.8725 | 0.0000 | 2.5407 | 0.0000 | 0.0001 | 0.0022 |  |  |
| Glycopeptide | vanA | 0.0000 | 73.0769 | 12.5000 | 0.0000 | 511.5088 | 16.3735 | 0.0000 | 9.3678 | 0.0000 | 0.0000 | 0.0242 | 0.0396 |  |
| Glycopeptide | vanC | 1.6393 | 34.6154 | 6.2500 | 0.0113 | 14.5323 | 0.0328 | 0.0000 | 0.0000 | 0.0000 | 0.0061 |  |  |  |
| Glycopeptide | vanD | 13.1148 | 30.7692 | 18.7500 | 0.8987 | 1.2920 | 0.7277 | 0.0000 | 0.0000 | 0.0000 |  |  |  |  |
| MLS | ErmB | 100.0000 | 100.0000 | 100.0000 | 131.1563 | 352.4491 | 252.3961 | 98.2866 | 243.6907 | 221.1663 | 0.0000 | 0.0063 |  |  |
| MLS | ErmF | 91.8033 | 80.7692 | 93.7500 | 25.3181 | 57.0598 | 26.7615 | 13.2668 | 11.7152 | 13.2071 | 0.0275 |  |  |  |
| MLS | ErmX | 55.7377 | 76.9231 | 68.7500 | 49.2545 | 27.0261 | 61.7317 | 0.5641 | 1.4620 | 5.1686 |  |  |  |  |
| MLS | ErmA | 0.0000 | 73.0769 | 12.5000 | 0.0000 | 125.0701 | 2.0893 | 0.0000 | 18.4416 | 0.0000 | 0.0001 | 0.0247 | 0.0406 |  |
| MLS | ErmT | 31.1475 | 69.2308 | 43.7500 | 2.2796 | 24.2449 | 3.5674 | 0.0000 | 1.3410 | 0.0000 | 0.0169 |  |  |  |
| MLS | lnuC | 95.0820 | 65.3846 | 87.5000 | 25.3208 | 13.8872 | 12.7354 | 9.7627 | 1.1263 | 6.7090 |  |  |  |  |
| MLS | ErmG | 96.7213 | 65.3846 | 87.5000 | 24.7135 | 13.8846 | 17.6770 | 11.6806 | 1.5065 | 19.8374 |  |  |  |  |
| MLS | MPH | 16.3934 | 57.6923 | 43.7500 | 4.4300 | 43.6728 | 134.0781 | 0.0000 | 0.6125 | 0.0000 | 0.0419 | 0.0041 |  |  |
| MLS | ErmQ | 55.7377 | 23.0769 | 37.5000 | 1.0993 | 2.1475 | 5.0121 | 0.5245 | 0.0000 | 0.0000 |  |  |  |  |
| Mupirocin | ileS | 63.9344 | 30.7692 | 37.5000 | 12.3871 | 9.0411 | 10.3287 | 6.3374 | 0.0000 | 0.0000 |  |  |  |  |
| Nucleoside | SAT-4 | 60.6557 | 84.6154 | 75.0000 | 2.6969 | 87.5040 | 5.7580 | 1.2193 | 31.8800 | 2.8238 | 0.0000 |  | 0.0252 |  |
| Peptide | bacA | 40.9836 | 80.7692 | 81.2500 | 4.3726 | 20.5365 | 34.7171 | 0.0000 | 7.6080 | 3.8382 | 0.0051 | 0.0008 |  | 0.0431 |
| Phenicol | catB3 | 1.6393 | 34.6154 | 0.0000 | 0.0135 | 32.2099 | 0.0000 | 0.0000 | 0.0000 | 0.0000 | 0.0171 |  |  |  |
| Phenicol | cat | 47.5410 | 34.6154 | 50.0000 | 1.4692 | 2.6594 | 8.1903 | 0.0000 | 0.0000 | 0.3173 |  | 0.0041 |  |  |
| Phenicol | catP | 67.2131 | 23.0769 | 50.0000 | 2.1664 | 1.7621 | 4.1989 | 1.0712 | 0.0000 | 0.3613 |  |  |  |  |
| Phenicol | catS | 32.7869 | 3.8462 | 43.7500 | 1.2901 | 0.0291 | 4.0502 | 0.0000 | 0.0000 | 0.0000 |  |  | 0.0103 |  |
| Polymyxin | pmr | 44.2623 | 80.7692 | 75.0000 | 13.9302 | 61.5260 | 91.6233 | 0.0000 | 25.6539 | 10.6803 | 0.0057 | 0.0009 |  |  |
| Sulfonamide | sul1 | 26.2295 | 80.7692 | 50.0000 | 3.5625 | 59.2288 | 67.4532 | 0.0000 | 2.4659 | 0.5064 | 0.0025 | 0.0039 |  |  |
| Sulfonamide | sul2 | 26.2295 | 57.6923 | 37.5000 | 3.1000 | 7.9136 | 26.7782 | 0.0000 | 1.0911 | 0.0000 |  | 0.0068 |  |  |
| Tetracycline | tetM | 90.1639 | 100.0000 | 81.2500 | 9.5315 | 82.4033 | 21.7767 | 6.1096 | 43.7750 | 7.2082 | 0.0000 | 0.0153 | 0.0045 |  |
| Tetracycline | tetW | 100.0000 | 80.7692 | 93.7500 | 108.2794 | 15.2633 | 104.8883 | 93.5363 | 10.2084 | 104.5944 | 0.0000 |  | 0.0000 |  |
| Tetracycline | tetQ | 100.0000 | 80.7692 | 93.7500 | 84.9120 | 87.5148 | 54.8649 | 66.0854 | 42.0218 | 41.4770 |  |  |  |  |
| Tetracycline | tetO | 100.0000 | 76.9231 | 93.7500 | 103.2342 | 33.8652 | 134.2528 | 99.3283 | 16.6749 | 118.7625 | 0.0000 |  | 0.0000 |  |
| Tetracycline | tet32 | 100.0000 | 65.3846 | 87.5000 | 37.0215 | 16.6498 | 44.1800 | 36.3711 | 8.2699 | 47.9742 | 0.0000 |  | 0.0005 | 0.0335 |
| Tetracycline | tetX | 68.8525 | 53.8462 | 50.0000 | 9.5824 | 31.2094 | 15.8629 | 2.3085 | 0.4323 | 0.4174 |  |  |  |  |
| Tetracycline | tetB(P) | 62.2951 | 19.2308 | 50.0000 | 1.3044 | 1.3420 | 2.1494 | 0.7009 | 0.0000 | 0.2476 |  | 0.0000 |  |  |
| Tetracycline | tet44 | 44.2623 | 15.3846 | 37.5000 | 1.8856 | 0.2085 | 1.4204 | 0.0000 | 0.0000 | 0.0000 | 0.0226 |  | 0.0232 |  |

Table S6. Correlation of the abundance of aminoglycoside-resistant genes with bacterial genus abundance ($rho>0.7$ and $p-value<e^{-3}$)

| correlation coefficient/  p-value | *Enterococcus* | *Morganella* | *Odoribacter* | *Parvimonas* | *Pediococcus* | *Porphyromonas* | *Prevotella* | *Proteus* | *Staphylococcus* |
| --- | --- | --- | --- | --- | --- | --- | --- | --- | --- |
| AAC(6')-Iae |  | 0.98/  0.0 |  |  |  |  |  | 0.99/  0.0 | 0.99/  0.0 |
| AAC(6')-Ic |  | 0.98/  0.0 |  |  |  |  |  | 0.99/  0.0 | 0.99/  0.0 |
| AAC(6')-Ie-APH(2'')-Ia | 0.77  /0.0 |  |  |  |  |  |  |  |  |
| AAC(6')-Ii | 0.89/  0.0 |  |  |  |  |  |  |  |  |
| AAC(6')-Iid |  |  | 0.78/  0.0 |  |  |  |  |  |  |
| AAC(6')-Iih |  |  |  |  | 0.95/  0.0 |  |  |  |  |
| aad(6) |  |  |  |  |  |  |  |  |  |
| aadA6_cluster |  |  |  |  |  |  | 0.72/  0.0 |  |  |
| aadA7 |  |  |  |  |  |  |  |  |  |
| ANT(3'')-Ii-AAC(6')-IId_fusion_protein |  |  |  |  |  |  | 0.72/  0.0 |  |  |
| APH(3')-IIb |  |  | 0.76/  0.0 | 0.76/  0.0 |  | 1.00/  0.0 |  |  |  |
